# Aerosol-Assisted Deposition for TiO_2_ Immobilization on Photocatalytic Fibrous Filters for VOC Degradation

**DOI:** 10.3389/fchem.2022.887431

**Published:** 2022-05-11

**Authors:** Sarka Drdova, Marianna Giannakou, Fuze Jiang, Luchan Lin, Deeptanshu Sivaraman, Rita Toth, Thomas Graule, Artur Braun, Jan Ilavsky, Ivan Kuzmenko, Jing Wang

**Affiliations:** ^1^ Institute of Environmental Engineering, ETHZ, Zürich, Switzerland; ^2^ Laboratory for Advanced Analytical Technologies, Swiss Federal Laboratories for Materials Science and Technology, Dübendorf, Switzerland; ^3^ Laboratory for Joining Technologies and Corrosion, Swiss Federal Laboratories for Materials Science and Technology, Dübendorf, Switzerland; ^4^ Laboratory for Building Energy Materials and Components, Swiss Federal Laboratories for Materials Science and Technology, Dübendorf, Switzerland; ^5^ Laboratory for High Performance Ceramics, Swiss Federal Laboratories for Materials Science and Technology, Dübendorf, Switzerland; ^6^ Advanced Photon Source, Argonne National Laboratory, Lemont, IL, United States

**Keywords:** aerosol-assisted deposition, titanium dioxide, photocatalysis, immobilization, VOC degradation

## Abstract

Atomization and spraying are well-established methods for the production of submicrometer- and micrometer- sized powders. In addition, they could be of interest to the immobilization of photocatalytic nanoparticles onto supports because they enable the formation of microporous films with photocatalytic activity. Here, we provide a comparison of aerosol-assisted immobilization methods, such as spray-drying (SD), spray atomization (SA), and spray gun (SG), which were used for the deposition of TiO_2_ dispersions onto fibrous filter media. The morphology, microstructure, and electronic properties of the structures with deposited TiO_2_ were characterized by SEM and TEM, BET and USAXS, and UV-Vis spectrometry, respectively. The photocatalytic performances of the functionalized filters were evaluated and compared to the benchmark dip-coating method. Our results showed that the SG and SA immobilization methods led to the best photocatalytic and operational performance for the degradation of toluene, whereas the SD method showed the lowest degradation efficiency and poor stability of coating. We demonstrated that TiO_2_ sprays using the SG and SA methods with direct deposition onto filter media involving dispersed colloidal droplets revealed to be promising alternatives to the dip-coating method owing to the ability to uniformly cover the filter fibers. In addition, the SA method allowed for fast and simple control of the coating thickness as the dispersed particles were continuously directed onto the filter media without the need for repetitive coatings, which is common for the SG and dip-coating methods. Our study highlighted the importance of the proper immobilization method for the efficient photocatalytic degradation of VOCs.

## 1 Introduction

In recent years, indoor air quality (IAQ) has been considered one of the main health risks. Particulate matter (PM), carbon monoxide (CO), nitrogen oxides (NOx), and volatile organic compounds (VOCs) belong to the typical indoor air pollutants (Huang et al., 2016). These pollutants originate from outdoor air infiltration and/or from indoor emission sources such as building materials, furniture, smoking, cooking, candles, and household products (Jones, 1999; Mamaghani et al., 2017). Modern buildings are constructed airtight to minimize the loss of thermal energy, and the supply of fresh air is controlled by the air-conditioning approach. Nevertheless, the fresh air supply is usually reduced in order to minimize the energy demand. Consequently, the pollutants are concentrated posing an elevated health risk to the occupants (Finnegan et al., 1984; Yu et al., 2009). The phenomenon called sick building syndrome is of environmental prevalence in modern buildings (Finnegan et al., 1984; Brown et al., 1994). The most effective way to control IAQ is either to use the strong dilution approach that implies high energy demand or to implement portable filters or filtration technology in central heating, ventilating, and air-conditioning systems (EPA, 2019). However, the filtration technology is traditionally designed to capture only the PM, while gaseous pollutants, such as VOCs, pass through the classical filtration procedure. Therefore, a technology capable of simultaneous removal of PM and VOCs is desirable to eliminate those pollutants in one step.

Recently, the integrated system of air filtration and purification of VOCs emerges as a promising alternative to the high-energy demanding dilution approach. Traditional air purification technologies consist of sorption materials for gases and odors. Although it is a commonly used method for VOC removal, this technique only transfers pollutants to another phase. Additionally, the sorption capacities pose a further limitation to this technology (Zhao and Yang, 2003; Yu et al., 2006). A heterogeneous photocatalytic oxidation (PCO) process is an alternative purification technique that offers superior properties, particularly the ability of VOC degradation into innocuous products (CO_2_ and H_2_O) through a series of reactions involving reactive oxidation species without a significant energy demand (Herrmann, 1999). Therefore, the implementation of PCO process for trace-level VOC removal from indoor air has gained attention in recent years (Jacoby et al., 1996). The most applied photocatalytic material is titanium dioxide (TiO_2_) due to its chemical benign nature, low cost, and medium-to-high efficiency at room temperature conditions (Huang et al., 2016). To implement the integrated system of PCO and filtration, an effective immobilization of photocatalyst nanoparticles plays a significant role. In general, the immobilization should meet requirements, such as good mechanical adhesion and chemical stability, minimal specific surface area reduction, sufficient light utilization, and an abundant contact area between the photocatalyst and pollutant (Han et al., 2012; Mamaghani et al., 2017). Dip-coating is a widely used method for glass substrate coatings due to its simplicity, low cost, and high film uniformity (Bouarioua and Zerdaoui, 2017). Nevertheless, the dip-coating method appeared to be not ideal for TiO_2_ immobilization on fibrous substrates because of the agglomerate formation resulting in a decrease of the PCO activity (Han et al., 2012).

The transformation of liquids containing precursors or solid particles into sprays and other dispersions is of great importance in several industrial processes. In recent years, the process of atomization that involves the formation of aerosol or suspension of small droplets in the gas phase has been studied for the immobilization of photocatalytic particles. More specifically, spray coating, in which the suspension of the photocatalytic particles is dispersed into tiny droplets, seems to be a promising alternative to dip-coating, providing good particle distribution and adhesion on fibers (Lefebvre and McDonell, 2017). Han et al. (2012) used a spray gun for the atomization and subsequent deposition of TiO_2_ photocatalytic particles onto a polyester fiber filter. They demonstrated higher degradation activity than the dip-coating method (Han et al., 2013). It could be attributed to the disintegration process that resulted in sub-micron-sized particles/agglomerates, as demonstrated in the recent study by Strob et al. (2018), in which two-fluid nozzle atomization was applied. The atomization process could prevent the formation of large agglomerates, and the size distribution mainly depended on the size of atomized droplets (Strob et al., 2018). In another study, Denny et al. (2010) applied the fluidized bed aerosol generator (FBAG) for TiO_2_ deposition on a glass fiber filter, and they compared this method with a manually loaded filter. They found that the FBAG method delivered more uniform distribution of TiO_2_ leading to more efficient UV light distribution, thus resulting in better photocatalytic performance. On the other hand, manual coating of TiO_2_ provided more significant aggregates and uncoated areas of fibers (Denny et al., 2010). There is no doubt that the elimination of agglomeration and maximization of light exposure are crucial requirements for efficient quantum conversion and the adsorption of pollutants (Mamaghani et al., 2017). However, the effect of the different atomization processes and dispersion levels on the photocatalytic activity of TiO_2_ has remained unclear, especially because the inter-study comparison is often difficult due to the different conditions used during the photocatalytic tests (e.g., type of catalyst, type and concentration of pollutants, or light source).

Here, we present a comparison of three aerosol-assisted immobilization of TiO_2_ photocatalytic particles in terms of the coating structure and adhesion and photocatalytic performances tested under uniform conditions. First, the spray-drying method was employed under different relative humidity (RH) conditions controlling the drying and deposition process as it has been shown that the humidity affected the morphology of airborne particles, their deposition, agglomeration, and filtration efficiency (Miguel, 2003; Wang et al., 2017). The spray-drying method formed compact structures. Here, we investigated the effect of different RH conditions on the TiO_2_ compact structures and their coating properties and ultimately on the photocatalytic activity. Second, the spray atomization with direct deposition of colloidal droplets was applied due to its capability to form uniform coating films. Both spray-drying and spray atomization were based on the injection principle using a commercial two-stream nozzle atomizer with an impaction section that resulted in a particle size distribution mainly below 1 µm. Third, a spray gun with direct deposition was used for the atomization and deposition as its atomization nozzle and droplet size were different from the atomizer. In addition, their photocatalytic performances were compared to the benchmark dip-coating method. In summary, we provided a comprehensive evaluation of aerosol-assisted deposition methods with a comparison to the benchmark dip-coating method. We propose a qualitative coating index for the evaluation of both photocatalytic and operational performances.

## 2 Materials and Methods

### 2.1 Materials

The titanium dioxide TiO_2_ (Degussa P25 Evonik) nanopowder with a particle size of 21 nm and measured specific surface area of 56 m^2^ g^−1^ (BET) was purchased from Sigma-Aldrich and used as a photocatalyst. The 1.5 wt% TiO_2_ suspension was prepared by adding the nanopowder into deionized water. The suspension was sonicated using an ultrasound bath (RK 255 H, Bandelin Sonorex) for 30 min and immobilized onto a glass fiber filter purchased from LydAir® with a basic weight of 68 g m^−2^ and 22% penetration (0.3 μm DEHS @5.33 cm s^−1^). The loading of TiO_2_ was set to 12 g m^−2^. The final mass loading of TiO_2_ was determined by weighing the dry filter before and after the immobilization process.

### 2.2 TiO_2_ Immobilization Techniques

**FIGURE 1:**
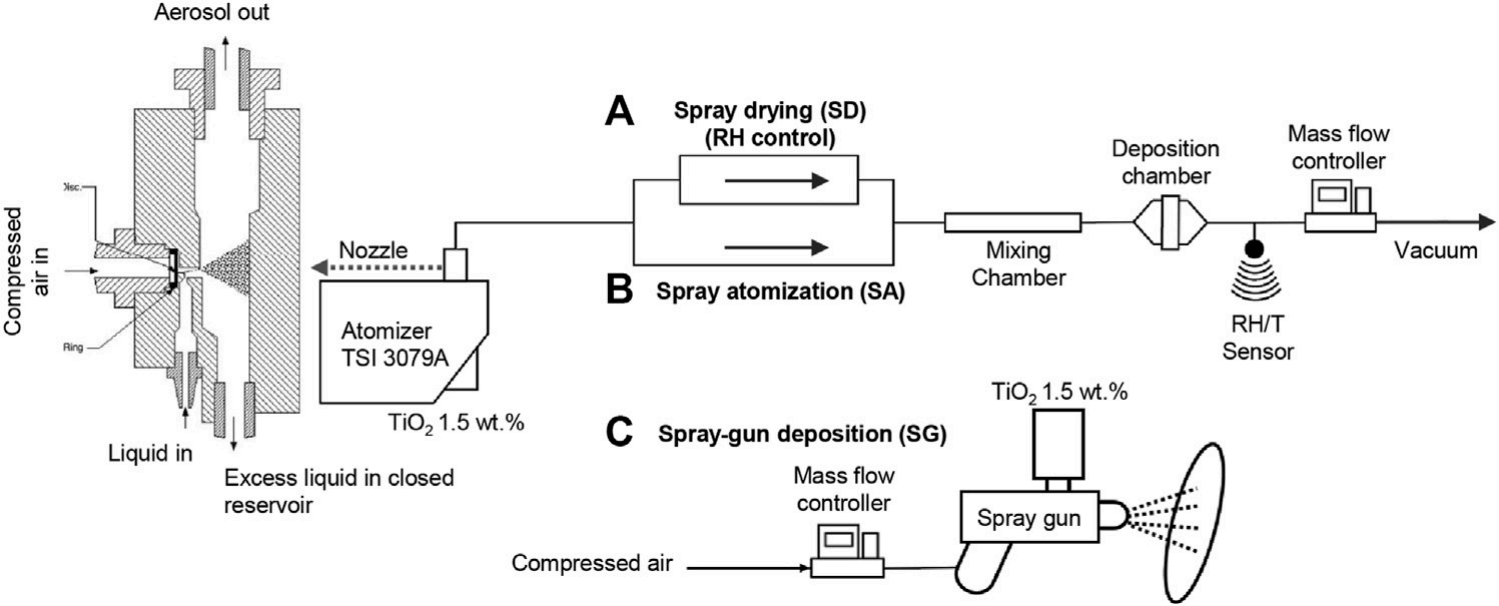
Schematic diagram of the atomization nozzle and aerosol-assisted deposition setups for **(A)** spray-drying (SD) with relative humidity (RH) control, **(B)** spray atomization, and **(C)** spray gun deposition.

Three aerosol deposition methods were employed to immobilize the TiO_2_ catalyst onto the glass fiber substrates. The suspension of TiO_2_ nanoparticles (NPs) was atomized using an atomizer from TSI (Model 3079A) and a spray gun (3.5 bar Aerotec 1.8 mm). First, the aerosol containing colloidal TiO_2_ droplets was generated by the TSI atomizer and exposed to different conditions. The relative humidity conditions of 30, 55, and 85%RH were controlled by a diffusion dryer and dry compressed air (Figure 1A), which is referred to as the spray-drying method (SD). The relative humidity and temperature during the deposition process were monitored using a Sensirion SHT7x sensor. Second, the atomized TiO_2_ droplets were directly deposited on the filter media corresponding to the spray atomization (SA) method (Figure 1B). In both cases, the depositions continued until the loadings of 12 g m^−2^ were obtained. The exact loadings of TiO_2_ were determined by the subtraction of the pristine filter mass from the mass of the filter after the coating and drying steps. The drying step was conducted under the ambient temperature, pressure, and relative humidity. The velocity of deposition, that is, the airflow velocity in the deposition chamber (Figures 1A and B), was 5 cm s^−1^ for both SD and SA methods. The size distributions of particles generated under the SD and SA conditions were measured upstream and downstream using a scanning mobility particle sizer and an aerodynamic particle sizer (Supplementary Figure S1). Third, the spray gun (SG) was connected to compressed air, and the sprayed TiO_2_ droplets were deposited with 50 L min^−1^ flow rate at a distance of 10 cm from the filter media. After the filter was covered with a layer of suspension, it was dried, and the process was repeated until the loadings of 12 g m^−2^ were obtained. In addition, a dip-coating method was used as a reference; a filter was dipped into the TiO_2_ suspension for 10 min, dried, and dipped again until the required loading was obtained.

### 2.3 Characterizations

The final crystalline phase of the produced samples was obtained with X-ray diffraction (XRD) on a PANalytical (X’Pert PRO) powder diffractometer equipped with a copper anode (Cu Kα radiation) and with X’Celerator detector. The time of a scan was set to 30 min, and the scan range was from 10 to 90°. The microstructures of as-prepared TiO_2_ nanoparticle clusters were characterized by transmission electron microscopy (TEM, JEOL2200FS), operating at the voltage of 200 kV. TiO_2_ nanoparticles were collected *in situ* after the exposure to different operational conditions on the ultrathin carbon film-supported Cu TEM grid (300 mesh, Ted Pella). To characterize TiO_2_ agglomerates deposited on the substrates, scanning electron microscopy (SEM) (Generation five Phenom ProX and FEI Quanta 650 FEG ESEM) was used. In order to prevent surface charging during imaging, the samples were sputtered with a 10-nm platinum layer. The size distribution and aspect ratio were estimated from the SEM images of particles deposited onto Nuclepore filters using ImageJ software.

Gas adsorption (BET) was used to measure the specific surface area, pore size, and volume distribution of materials through nitrogen adsorption (Micrometrics), and the software (MicroActive for 3Flex, Version 4.04, Micromeritics) was used for the computation of results. Nitrogen adsorption and desorption isotherms were measured at liquid nitrogen temperature (−196°C) on a Micromeritics 3Flex instrument after degassing the samples at 2.5 × 10^–2^ mmHg and 100°C for 20 h. The specific surface areas were calculated from seven data points in the linear range of P/P_0_ between 0.05 and 0.3 using the Brunauer–Emmett–Teller (BET) method. The average pore width and total pore volume were estimated from the Barrett–Joyner–Halenda (BJH) model assuming cylindrical pores and from the non-local density function theory (NLDFT) assuming slit pore dimensions.

Ultra-small-angle X-ray scattering (USAXS) and small-angle X-ray scattering (SAXS) measurements were carried out using the 9ID USAXS instrument at Advanced Photon Source, Argonne National Laboratory (Ilavsky and Jemian, 2009; Ilavsky et al., 2013). The combined q range was between 1.10^−4^ Å^−1^ and 1.3 Å^−1^, where q was equal to 4π/λ sin(θ) (*λ* was the wavelength, and *θ* was ½ of the scattering angle). The X-ray energy was 21 keV (*λ* = 0.5895 Å). The X-ray photon flux was ≈5∙10^12^ mm^−2^s^−1^. The samples were prepared by the deposition of powders in the center of a steel washer (outer diameter 8.55 mm and inner diameter 1.5 mm), which was closed on both sides with a scotch tape. The mass of powders was measured using a microbalance. Depending on the sample, the mass was between 31 and 50 mg.

Thermogravimetric analysis (TGA) was performed on a Perkin Elmer TGA 8000 under a constant flow of nitrogen of 40 ml/min. First, the samples were heated from 30 to 120°C at the rate of 20°C/min and held for 10 min to remove the physically adsorbed water. After that, the temperature was elevated from 120 to 500°C (at 20°C/min) to determine the amount of adsorbed hydroxyl groups. A Thermo Scientific ESCALab 250Xi XPS (Thermo Fisher Scientific) with 200-W monochromated Al Kα radiation was used to perform X-ray photoelectron spectroscopy (XPS) analysis. All binding energy values were calibrated by fixing the C1s pattern to 285 eV. Gaussian–Lorentzian components were used for peak deconvolutions after a Shirley background subtraction. The relative area ratio of XPS peaks was used for sample comparison.

Diffuse reflectance spectroscopy was used to characterize the optical properties of the coated filters. The UV-Vis-NIR Spectrophotometer (UV-3600, Shimadzu) was used to measure the transmittance and reflectance of light using three detectors (photomultiplier tube (PMT), In-GaAs, and cooled PbS) that enabled the measurement over the ultraviolet, visible, and near-infrared regions (300–1,500 nm). A high-performance double monochromator was used for the measurements. A high-speed USB pressure sensor (PX409 Series Pressure Transducer, Model No. PX409-10WDWUUSBH, Omega) with a micro-machined silicon design and the viewing software (Digital Transducer Application, Omega) were used to measure the pressure drop of coated and uncoated glass fiber filter to determine the change in performance. The flow velocity applied to the samples was 5 cm s^−1^. The flow conditions in the system provided laminar flow allowing using Darcy’s law for the determination of permeability of the filter media before and after immobilization of TiO_2_ particles. The stability of TiO_2_ NPs on the coated filter was tested under high-stress conditions. The procedure was adapted from Wohlleben et al. (2017). A square piece of 2 cm × 2 cm of each sample was immersed in 6 ml of the solution prepared by adding 5.7 ml glacial acetic acid in 500 ml of reagent water and 64.3 ml of 1 M NaOH, which was diluted to a volume of 1 L. The solution with the sample was held in a 10 ml cylindrical glass container and sonicated in an ultrasonic bath (RK 255 H, Bandelin Sonorex) for an hour. The resulting specimens were sonicated for an extra half hour after the samples were removed in order to eliminate the agglomeration and sedimentation of particles before absorbance analysis using a UV-Vis spectrometer.

### 2.4 USAXS Data Analysis

The X-ray scattering curves obtained from ultra-small scattering measurements were analyzed using the Unified fit in the “Irena” software package (Ilavsky and Jemian, 2009; Ilavsky, 2018). The Unified fit uses the equation for the scattered intensity as derived by Beaucage (1995); Beaucage (1996); Beaucage et al. (1997):

$$I_{i}\left(q\right)=\;G_{i}\cdot e^{-\frac{q^{2}\cdot R_{g}^{2}}{3}}+\;e^{-\frac{q^{2}\cdot R_{g\_co}^{2}}{3}}\;B_{i}\;{\left\{\frac{{\left(erf\left(\frac{q\cdot R_{g}}{\sqrt{6}}\right)\right)}^{3}}{q}\right\}}^{P_{i}}.$$ (1)

The relevant fit parameters are the radii of gyration *R*_*g*_, the exponent of decay *P*_*i*_, and the two constants *G* and *B*. The Unified fit splits the scattering curve into different structural levels with a Guinier regime reflecting the structure’s radius of gyration and a power law regime, which displays the type of the structure (Londoño et al., 2018). The first structural level describes the primary particle radius of gyration *R*_*g1*_, which is the mean square distance from the center of electron density of a particle (in analogy to the momentum of inertia in mechanics) and is converted to the diameter of spherical primary particles as:

$$D_{pp}=2\cdot \sqrt{\frac{5}{3}}\cdot R_{g1}.$$ (2)

From the slope of scattering intensity decay, fractal dimension D_f_ can be assessed as the power law regime following I(q) ∼ q^Df^, which allows investigation of the structure of the aggregates. The radius of gyration (R_g2_) of the second structural level was used for the determination of the radius and diameter of agglomerates using Eq. 2 (Braun et al., 2004). The degree of aggregation is characterized by a parameter *z* that is directly obtained from a constant *G* for primary particles (*G*_*pp*_) and aggregates (*G*_*agg*_) as $z=G_{agg}/G_{pp}$ (Hyeon-Lee et al., 1998). The radius of gyration for aggregates is determined from the diameter of primary particles, aggregation number z, and fractal dimension as:

Rgagg= (Dpp2⋅z2Df(1+2Df)⋅(2+2Df))12. (3)

### 2.5 Photocatalytic System and Evaluation of Photocatalytic Activity

**FIGURE 2:**
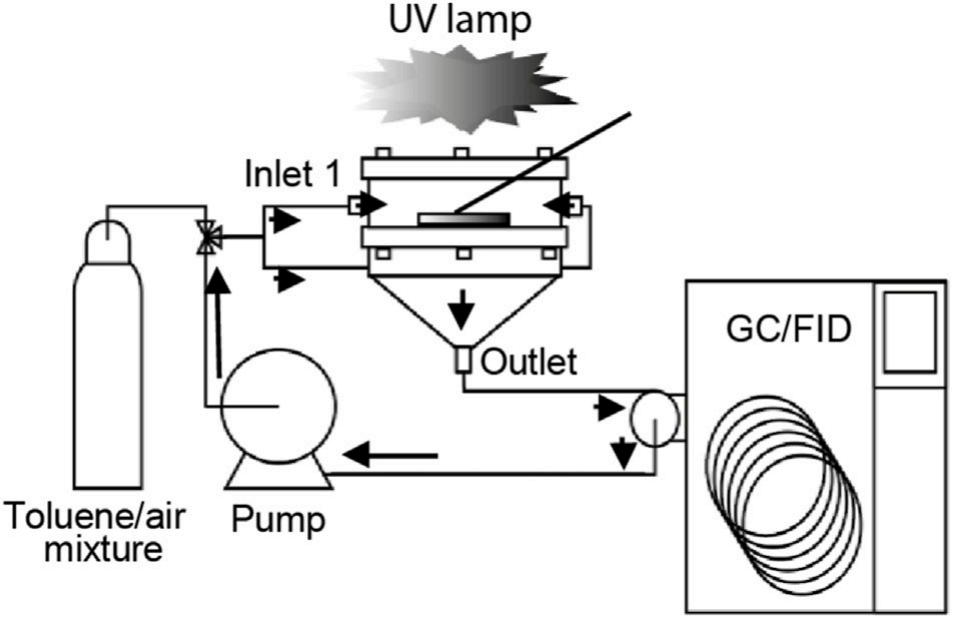
Schematic diagram of the toluene degradation setup.

The evaluation of catalytic degradation activity was performed in a stainless steel reactor of the filter holder type, which was designed and assembled according to a previous study (Zhao et al., 2020). The filter coated with the catalyst was installed between the upper and bottom parts of the photoreactor and supported by a stainless steel mesh. The upper part consisted of a boron silicate glass cover allowing the filter illumination using a lamp placed horizontally above the reactor. Toluene mixed with air was introduced into the reactor chamber through two opposite inlets facilitating better flow distribution. The concentration of toluene was monitored by gas chromatography GC-2014 (Shimadzu) equipped with an Rtx-Wax separation column and an FID-2014 (Shimadzu) detector (Figure 2). The initial toluene concentrations were 5, 20, and 40 ppm. After the satisfactory introduction of the toluene–air mixture into the reactor, the system was closed, and the gas was circulated until reaching a steady-state condition. The temperature and relative humidity inside the reactor were approximately 22°C and 20% RH, respectively. Several control tests, such as adsorption or photolysis evaluation, were performed (Supplementary Figures S2A–C). The samples were irradiated using six UV BLB lamp bulbs (368-nm) emitting an intensity of 0.6 mW cm^−2^ (Supplementary Figure S2D).

The photodegradation rate of toluene was evaluated following the Langmuir–Hinshelwood (L-H) kinetic model (Coronado et al., 2013). The initial degradation rate (r_0_) is observed to be a function of the initial concentration (C_0_). A linear plot of r_0_^−1^ versus C_0_^−1^ allowed us to obtain the L-H rate constant *k* and Langmuir adsorption constant *K*_*L-H*_ as follows:

$$r_{0}=\frac{k_{L-H}K_{L-H}C_{0}}{1+K_{L-H}C_{0}}\;or\;\frac{1}{r_{0}}=\frac{1}{k_{L-H}K_{L-H}}\frac{1}{C_{0}}+\frac{1}{k_{L-H}}.$$ (4)

For diluted systems, where the concentration is lower than 10^−3^ M (1,000 mmol m^−3^), *KC*_*0*_ becomes much lower than 1, and the reaction follows an apparent first-order kinetic model (Herrmann, 1999). Therefore, a simplified pseudo-first-order kinetic model was applied for the degradation of toluene as (Liu et al., 2005) follows:

$$ln\frac{C_{0}}{C}=k_{1}\cdot t.$$ (5)

where *C*_*0*_ is the initial concentration of toluene, *C* is the concentration of toluene at the specific reaction time *t* in min, and *k*_*1*_ is the rate constant of the pseudo-first-order model in min^−1^. The reaction constant can be obtained from the slope of *lnC*_*0*_ */C* versus *t* plot. When R^2^ is larger than 0.95, the experimental data are considered to be in good agreement with this model (Fox and Dulay, 1993).

## 3 Results and Discussion

### 3.1 Characterization of TiO_2_ Coatings

#### 3.1.1 X-Ray Diffraction

**FIGURE 3:**
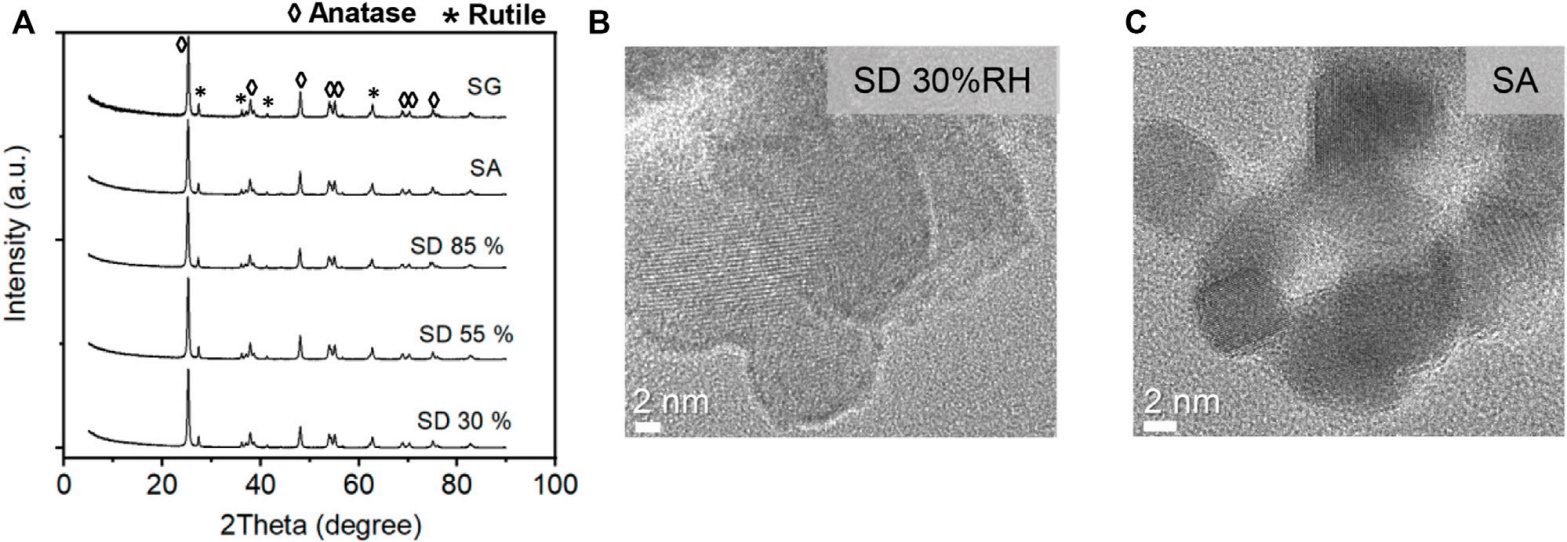
**(A)** X-ray diffraction patterns of TiO_2_ immobilized with different aerosol-based deposition methods and HRTEM images of TiO_2_ nanoparticles deposited with **(B)** spray-drying and **(C)** spray atomization methods.

The X-ray diffraction (XRD) patterns of generated TiO_2_ powder samples and glass fiber (GF)-coated samples prepared by spray-drying (SD), spray atomization (SA), and spray gun (SG) methods were investigated to determine any change in the crystallinity due to the immobilization process. The XRD pattern of the as-made samples showed strong Bragg reflections except for a slight change in intensity (Figure 3A), which indicates that the crystalline phase did not alter significantly from the original material after the preparation process. The XRD and HRTEM analysis (Figures 3B and C) confirmed the mixture of anatase and rutile phases.

#### 3.1.2 Structure of TiO_2_ Agglomerates

The structure of TiO_2_ agglomerates prepared by SD, SA, and SG methods were investigated visually using TEM and SEM techniques and characterized by USAXS analysis.

**FIGURE 4:**
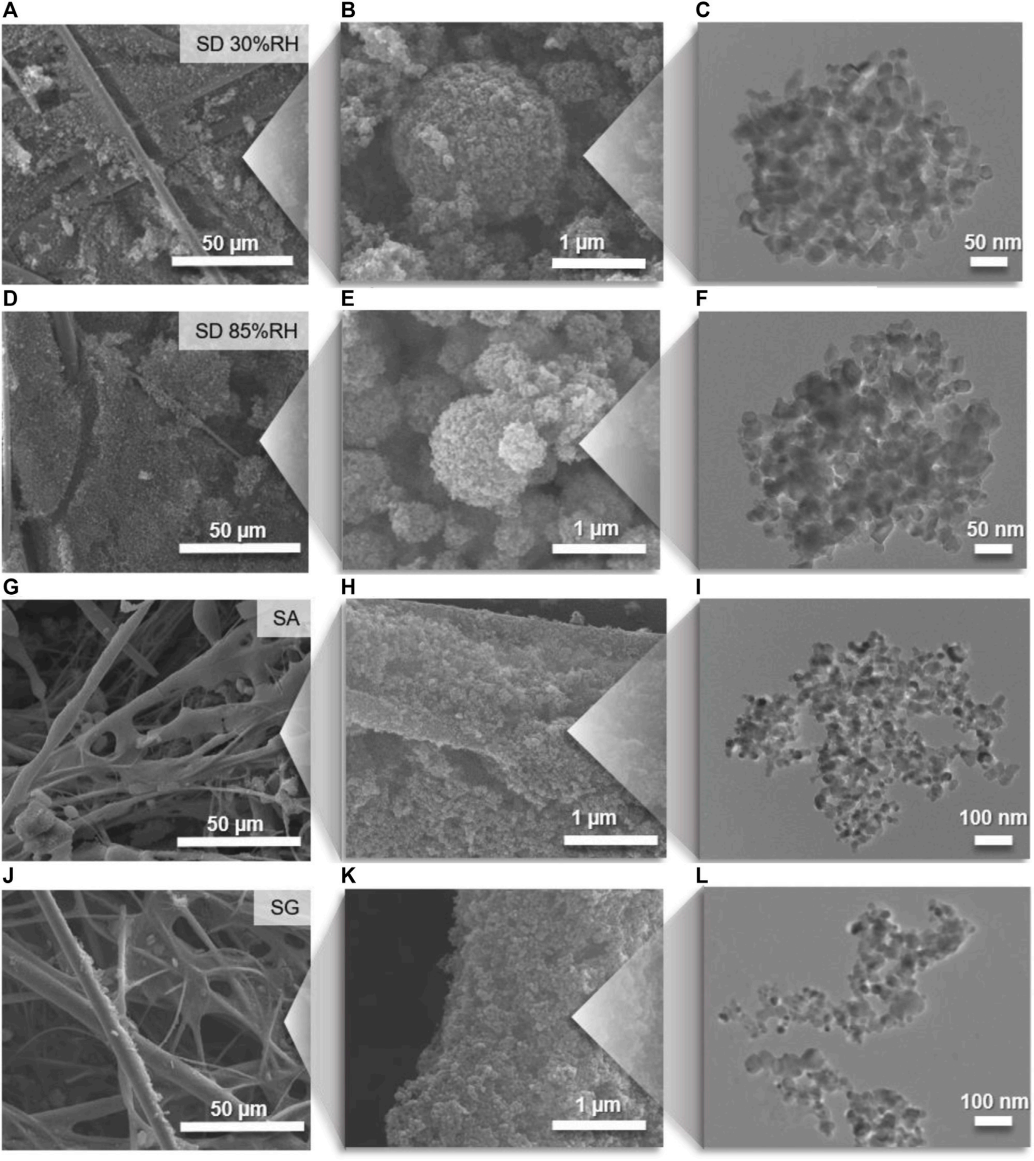
SEM images of coated filters and higher magnification of TiO_2_ agglomerate structures coupled with TEM images of typical individual agglomerates prepared by **(A–C)** spray-drying (SD) at 30%RH and **(D–F)** SD at 85%RH, **(G–I)** spray atomization (SA), and **(J–L)** spray gun (SG) deposition methods.

**FIGURE 5:**
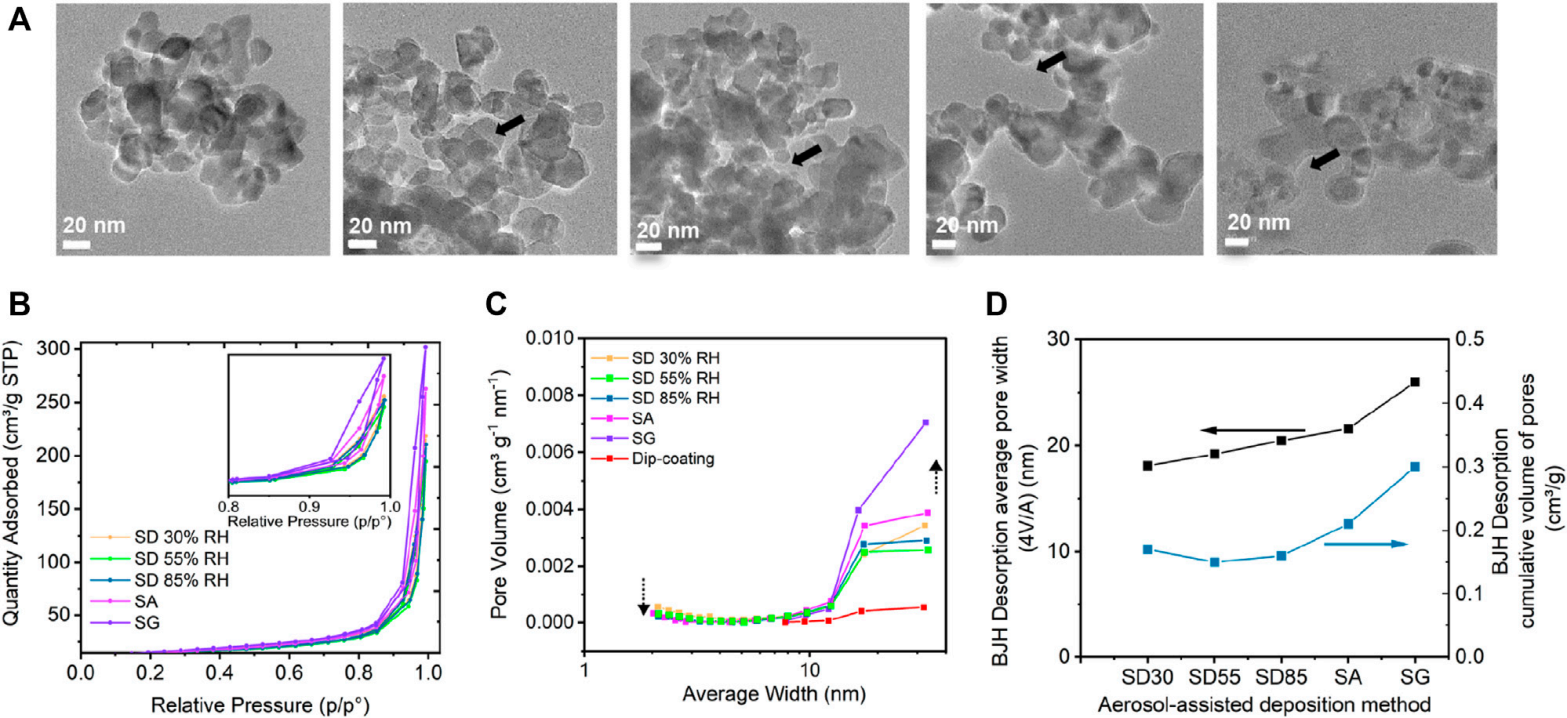
TEM microscopic characteristics of atomized TiO_2_ particles using spray-drying (SD) at **(A)** 30, 55, and 85%RH, spray atomization (SA), and spray gun (SG) deposition methods, with a focus on the compactness of the agglomerates. **(B)** BET nitrogen adsorption–desorption isotherms for different samples. **(C)** BJH pore size distribution and **(D)** effect of the deposition procedure on averaged pore width and cumulative volume of pores.

The SEM (Figure 4) and TEM (Figure 5) images showed that the TiO_2_ NPs are agglomerated, and their structures differ significantly for the different coating methods. After the drying of atomized TiO_2_ particles (SD methods), the particles formed round and compact agglomerates, which accumulated mainly between and on the top of the GF fibers. The resulting structures of agglomerates were similar for all examined samples prepared by SD at 30, 55, and 85%RH conditions (Figures 4A–F) and remained round and compact after the deposition onto the substrate (Figures 4A and D), which corresponds to the hard agglomerate behavior. On the other hand, the direct deposition of atomized particles in the form of droplets by SA and SG methods provided open and fractal-like agglomerates (Figures 4G–L) after the drying process on the filter. The large and fractal structures were accompanied with small fragments consisted of several primary particles (Supplementary Figure S3). The small fragments indicated the soft agglomerate behavior, that is, the bound energies between the constituent primaries were broken upon the impaction on the substrate, which was related to the presence of water (Froeschke et al., 2003; Ihalainen et al., 2014). This behavior allowed for the formation of a uniform layer of TiO_2_ NPs on the glass fiber surface resembling the original structure of the GF filter. Consequently, the different deposition methods affected the compactness and pore sizes of the immobilized TiO_2_ (Figure 5A). While SD at low RH conditions provided high compactness with the negligible presence of pores, the increase in RH resulted in the formation of a more open structure. For SA and SG methods, the pores were large and prevalent (Figure 5A).

The observation from TEM and SEM is further accompanied by the nitrogen sorption isotherm analysis using the BET method in order to determine the pore size and distribution. The shape of the isotherms represents Type-IV with an H3 hysteresis loop ranging from 0.85 to 1 P/P_0_ relative pressure (Figure 5B), which is the characteristic for agglomerated particles, and it indicates the mesoporous nature of the samples with non-uniform slit-like pore structures (Sing and Williams, 2004; Chen et al., 2014; He et al., 2015). As reported by He et al. (2015), the shape of the H3 hysteresis originates from a broad pore size distribution, which was analyzed using BJH (Figure 5C) and NLDFT (Supplementary Figure S4A) models. NLDFT model uses a carbon slit pores model as an approximation to the slit pore geometry that was determined for our samples. This well-established model has been applied for the characterization of mesoporous titanium with enhanced photocatalytic activity by Jaafar et al. (2015) and lately, for the characterization of titanium dioxide by Peng et al. (2020) showing the pore size distribution from 1.5 to 30 nm. It should be noted that this model works well only for the particle size distribution below 100-nm (Ravikovitch et al., 1998). Therefore, the larger pore sizes observed from TEM images are excluded from the pore size distribution analysis. Our results revealed broad pore size distributions in the range between 2 and 60 nm (Supplementary Figure S4A). The resulted distributions demonstrated that some of the smaller pores below 10 nm disappeared or were less abundant for the SD 85%RH sample than the lower RH samples while larger pores in the range from 13 to 60 nm were ample. The SA sample showed the largest number of pores from 3 to 60 nm, and the SG sample exhibited the largest number from 13 to 58 nm (Supplementary Figure S4A). This is supported by the total volume of pores and by the average pore width (Figure 5D) determined by a standard BJH model (Luisa Ojeda et al., 2003). The BET surface area results follow the trend of average pore volume (Supplementary Table S1). From SD 30%RH to SD 55%, the BET surface area decreased from 52 m^2^ g^−1^ to 47 m^2^ g^−1^. For SD 85%RH, SA and SG, the BET increased steadily up to 54 m^2^ g^−1^. The porosity determined from the analysis of density confirmed very low porosity of SD 30%RH and higher porosity of SG sample (Supplementary Table S1).

**FIGURE 6:**
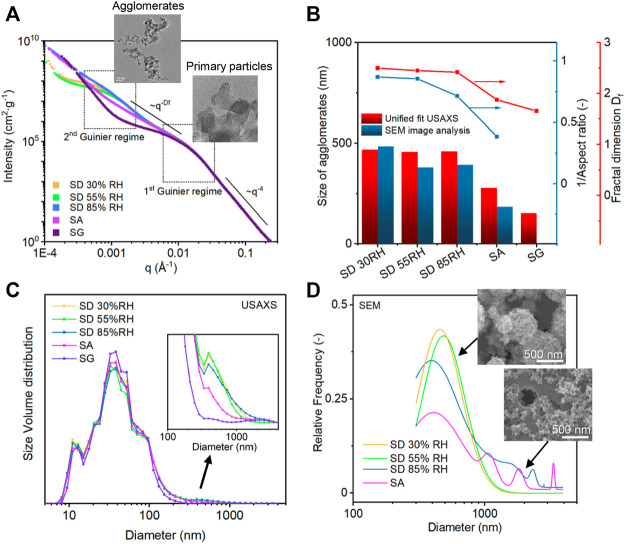
**(A)** USAXS scattering curves of the TiO_2_ powder samples prepared by different aerosol-assisted deposition methods divided into four regimes. **(B)** Comparison of the count median diameter (CMD) determined from SEM size distribution and diameter calculated from R_g_ obtained from the Unified fit model (left y-axis) and reciprocal aspect ratio analysis and fractal dimensions determined from the SEM image and from the regime three of Unified fit, respectively (right y-axis). **(C)** Size distribution of aggregates determined by the Irena size distribution fitting tool. **(D)** Size distribution determined from SEM images.

Ultra-small–angle X-ray scattering (USAXS) was performed on the samples prepared with different aerosol-assisted deposition methods. The USAXS curves were fitted by the Unified fit (Ilavsky and Jemian, 2009), which divided the complex scattering patterns into four regions that contained specific information about the structures of particles and aggregates (Figure 6A and Supplementary Figure S5). The first region of the high q range (q > 0.016 Å^−1^), where the level 1 fit was performed, is known as the Porod region, reflecting the structure of primary particles. All the samples showed the same Porod law with a constant slope of q^−4^, indicating a smooth surface of primary particles. This region was followed by two Guinier shoulders in the range 0.01 Å^−1^ < *q* < 0.02 Å^−1^ and 0.0004 Å^−1^ < *q* < 0.005 Å^−1^. All the samples shared the first Guinier shoulder (Figure 6A), which corresponded to an object length of around 28 nm (according to the relation L = 2π/q). We assigned this length to the primary particle diameter of TiO_2_ P25, which is in agreement with TEM analysis and reflects the properties of the original TiO_2_. Accordingly, the Unified fit in this region was adjusted using predefined parameters correlated with the known primary particle size and BET surface area of TiO_2_ (Supplementary Figure S6A). The shape of the second Guinier shoulder varied for the different deposition methods and corresponded to the structure of TiO_2_ agglomerates with the object length around 400 nm. This Guinier shoulder was more pronounced for SD samples, while there was no additional Guinier shoulder for SA and SG samples accounting for the change in the agglomeration characteristics (Figure 6A) (Hyeon-Lee et al., 1998). The radius of gyration of the second Guinier level was therefore assigned to the agglomerated structures of TiO_2_ NPs, and their size was calculated assuming spherical shape as D_agg_ = 2 (5/3)^1/2^R_g_. The results showed that the diameters of the agglomerates were 517, 430, and 403 nm for SD methods, and it decreased to 279 and 234 nm for SA and SG methods, respectively (Figure 6B). These results correlated well with the sizes determined by the SEM analysis of agglomerates collected on a Nucleopore filter within a short deposition time (note that smaller objects below 300 nm were excluded from the size distribution analysis for better comparison in this size range). The decrease of SA and SG size and volume in this range was attributed to the change in the agglomerate structure due to the presence of TiO_2_ NPs in the water droplets. The final structure of agglomerates can be obtained from the region located between the two Guinier shoulders (0.001 < *q* < 0.02 Å^−1^). The slope of this region characterized as the power law of I(q) ∼ q^−Df^ provides information about the fractal dimensions of agglomerates (Figures 6A,B). As expected from the TEM and SEM visual inspections, the resulted fractal dimension of D_f_ = 2.7 and 2.6 for SD 30%RH and 55%RH deposition methods corresponded to the mostly spherical structures, whereas it decreased for SD 85%RH, SA, and SG methods (Df = 2.4, 2.1, and 2.0, respectively) confirming the formation of more open and fractal-like structures. The results of D_f_ were compared and agreed well with the reciprocal values of aspect ratios (AR) determined from the SEM analysis (Figure 6B), which was performed on the agglomerates deposited on Nucleopore filters. The resulted D_f_ values together with other Unified fit parameters, such as the aggregation degree and size of the primary particles (R_g1_), were additionally used for the determination of agglomerate sizes according to Eq. (3) (Supplementary Figure S6). Due to different sampling and weighing procedures, the USAXS results differed slightly from SEM analysis, but both analyses demonstrated the same trend.

To further analyze the scattering objects, a size distribution was obtained for each USAXS curve using a maximum entropy method in the Irena tool (Jemian et al., 1991) and compared with the size distribution in the range larger than 300 nm obtained from SEM images (Figures 6C and D, and Supplementary Figure S7). The USAXS results displayed multimodal distributions with a high volume fraction at around 30 nm and two low volume fractions at larger sizes (Figure 6C). The first peak represented the size distribution of TiO_2_ primary particles with the main contribution of around 30 nm and a significant contribution from the smallest NP size of around 13 nm (confirmed by TEM), which was in accordance with previous studies (Turković et al., 1998; Körösi et al., 2007). The contribution of middle sizes between 50 and 100 nm was related to the internal structures of agglomerates, while the second peak at the size of around 400 nm corresponded to the external sizes of agglomerates (Figure 6C, magnified part). The volume fraction in this size range decreased gradually for SD 85%RH, SA, and SG. This was attributed to the gradual disappearance of round and compact agglomerates followed by increasing fragmentation and de-agglomeration effect due to the increasing content of water and water droplets (Supplementary Figure S8). On the other hand, the additional small fraction of the size larger than 1,000 nm for SA and SG was assigned to the reorganization and agglomeration on the substrate.

**FIGURE 7:**
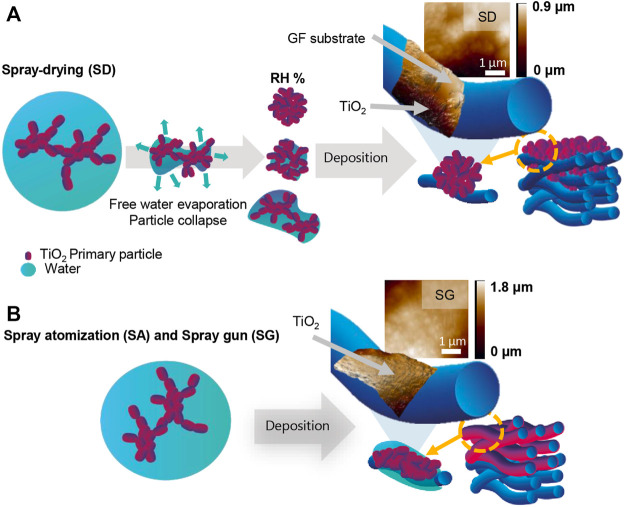
Schematic illustration of deposition processes under **(A)** spray-drying (SD), **(B)** spray atomization (SA) and spray gun (SG) conditions with a fiber surface image analysis using atomic force microscopy (AFM) to demonstrate the deposition mechanism and the TiO_2_-coating coverage uniformity.

In summary, the morphology of the coated filters depends on the deposition conditions and the level of fragmentation after the impaction on the substrate (Figure 7 and Supplementary Material S1.1). The presence of water droplets contributes significantly to the fragmentation or de-agglomeration, which could occur during the spreading and splashing of the atomized droplets onto the solid surface, followed by spontaneous evaporation even though the deposition velocity was rather low (Froeschke et al., 2003; Ihalainen et al., 2014). This produced inter- and/or intra-molecular interactions among the suspended nanoparticles and substrate forming a disordered structure on the surface of the substrate, while certain properties of the original TiO_2_, such as the crystalline structure and primary particle diameter, were unaffected by the deposition method.

#### 3.1.3 Hydration of Deposited TiO_2_ Nanoparticles Using TGA and XPS Techniques

Apart from the structure of the coating, water adsorption is another crucial factor that is known to be essential in photocatalytic processes. In the photocatalytic process, the band gap energy is exceeded, which promotes an electron from the valence band to the conduction band. The resultant electron–hole pair participates in chemical reactions generating the reactive species, such as hydroxyl radicals and super-oxide ions. These species oxidize VOCs adsorbed on the catalyst surface (Jacoby et al., 1996; Zhao and Yang, 2003; Liu et al., 2021). Water adsorbed on the photocatalyst provides additional hydroxyl radicals, which are considered the main reactant for the oxidation process. Therefore, the degree of TiO_2_ surface hydration depends on the surface structure and the nature of the adsorption mechanism (Benkoula et al., 2015; Wu et al., 2017; Orlando et al., 2019). In order to evaluate the level of TiO_2_ hydration after the aerosol-assisted deposition processes, the content of hydroxyl groups and adsorbed water were assessed using TGA and XPS techniques.

**FIGURE 8:**
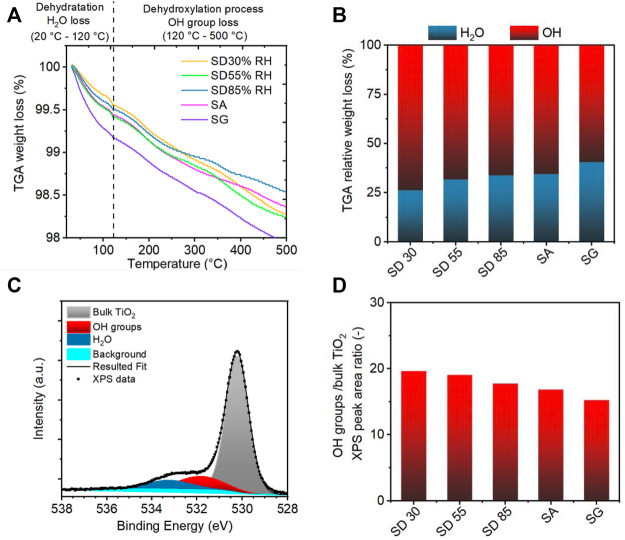
**(A)** Thermogravimetric analysis (TGA) of TiO_2_ NPs deposited with different aerosol-assisted deposition methods. **(B)** TGA relative weight loss of adsorbed water (blue) and hydroxyl groups (red). **(C)** X-ray photoelectron spectroscopy (XPS) spectra of the O 1s core level of the SD 30%RH sample with peak fitting for oxygen originated from bulk TiO_2_, OH groups, and adsorbed water. **(D)** Ratio of OH groups and bulk TiO_2_ peak area for samples prepared with different deposition methods.

TGA is a simple and fast technique that allows the determination of TiO_2_ surface OH groups (Figure 8A) (Di Paola et al., 2014; Wu et al., 2017; Cui et al., 2020). In particular, the loss of weight below 120°C is assigned to the loss of physically adsorbed water, whereas the loss of weight within the range of 120–300°C and 300–500°C corresponds to weakly and strongly bonded OH groups, respectively (Wu et al., 2017). The total weight losses were similar for all the samples at around 1.7 wt% ± 0.2 wt%. The chemisorbed water represented as weakly and strongly bonded OH groups was the main component of the total weight loss at 500°C (around 60–70% of total loss) (Figure 8B). The ratio of physically adsorbed water increased with the increasing amount of water present during the preparation process, for example, higher RH conditions during SD and deposition of colloidal droplets during SA and SG methods (Figure 8B). In contrast, the fraction of OH groups decreased (Figure 8B). Our results showed that the SD with lower RH conditions resulted in the dissociative adsorption of water, whereas more physically bounded water remained on the TiO_2_ surface during the higher RH conditions and in SA and SG methods.

XPS has been reported as a useful tool for the understanding of TiO_2_ NP surface properties. Several studies focused on the hydration process and water dissociation on TiO_2_ NPs or TiO_2_ nanocrystals (Perron et al., 2007; Benkoula et al., 2015; Wu et al., 2017). There are three main water-related components of the O 1s spectrum that have been used to probe the surface composition with respect to the adsorbed water, Ti–O bulk oxide, bridging OH groups, and terminal OH groups. In our XPS O 1s spectra (Figure 8C), the main peak at 530.2 ± 0.05 eV BE (binding energy) is assigned to lattice oxygen in the surface TiO_2_, the second peak at 531.8 ± 0.04 eV BE to the adsorbed OH groups, and the third XPS pattern at 533.2 ± 0.07 eV BE corresponds to adsorption of water on the TiO_2_ surface (H_2_O peak in Figure 8C). The third peak is, however, affected by desorption under ultrahigh vacuum conditions of XPS and could not be directly assigned to the hydration level of samples (Perron et al., 2007; Benkoula et al., 2015). To evaluate the spectra, the binding energy shift of OH groups and H_2_O from the lattice O was measured and determined as 1.6 and 3.0 eV, respectively, which is consistent with reported values (1.2–1.6 and 3.3–3.6 eV) (Perron et al., 2007; Benkoula et al., 2015) for TiO_2_ nanopowders. In analogy to TGA results, the evaluation of the relative abundance of OH groups to the bulk TiO_2_ oxygen revealed that the ratio of OH groups decreased with the presence of water during the aerosol-assisted deposition (Figure 8D). Our findings showed that dissociated and molecular adsorbed water existed on the TiO_2_ NP surface after the deposition. It has been reported that water dissociation during the adsorption on TiO_2_ rutile and anatase surfaces is favored at lower water coverage, which is enhanced by the presence of surface defects, while higher water coverage results in the adsorption of water molecules (Henderson, 1996; Blomquist et al., 2008; Shen et al., 2019). Accordingly, an increased amount of molecular water was observed for SD under higher RH conditions, SA, and SG methods, owing to the higher water coverage during the deposition (Walle et al., 2011; Orlando et al., 2019).

#### 3.1.4 Optical Properties of TiO_2_-Deposited PCO Filters

**FIGURE 9:**
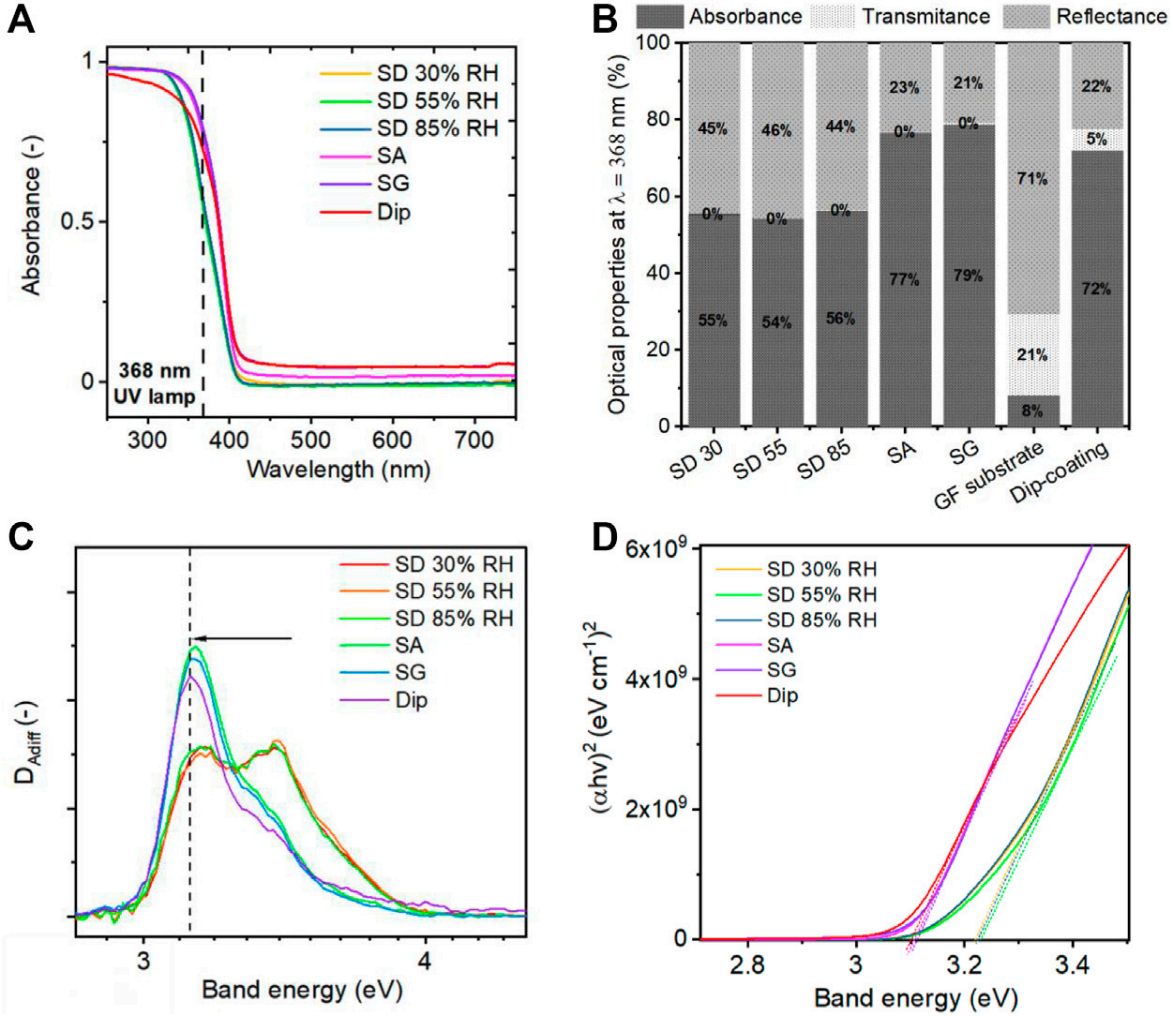
**(A)** Absorbance spectra of TiO_2_ NPs coated on glass fiber filters with different aerosol-assisted deposition methods. **(B)** Transmittance, reflectance, and absorbance at the wavelength of 368 nm, which was used in this study during the photocatalytic tests. **(C)** First derivative (D_Adiff_) analysis of adsorption spectra for band gap determination with fitted values of transition energies. **(D)** Tauc plot for band gap determination.

The light propagation and absorption properties of a photocatalyst have been shown as crucial parameters for the effective activation of the photocatalytic process (Melcher et al., 2017). Therefore, light propagation over the immobilized filters was examined using the diffuse reflectance technique. The absorption spectra of TiO_2_ NPs coated on the glass fiber filter media showed slightly different optical behaviors for different deposition methods (Figure 9A). A slight red shift in the absorption spectra was observed for the samples prepared under SA and SG conditions. This has been recently observed by Melcher et al. (2017) for deagglomerated TiO_2_ particles. The red shift affected the shares of absorption, transmittance, and reflectance at the used wavelength (368-nm). The results showed that the UV light was more efficiently utilized by the samples prepared under SA and SG conditions, where only about 20% of light was reflected (Figure 9B). On the other hand, the samples prepared by the SD method showed more than 40% of reflectance. The high amount of the reflected light originated from the structure of the coated filters and from the size of the agglomerated particles. It has been reported that the round and more compact agglomerated particles scatter light more significantly, which is unfavorable for the photocatalytic process (Melcher et al., 2017; Pellegrino et al., 2017). The second reason for the high reflectance was the formation of a cake on the top of the filter resulting in the inability for the light to penetrate deeper into the filter structure.

We used Tauc’s analysis and the first derivative analysis of the adsorption spectra to determine the adsorption edges and optical band gaps (Lin et al., 2006; Michalow et al., 2012; Flak et al., 2013). The first derivative analysis of the Tauc plot followed by peak fitting (Supplementary Figure S9) revealed two transition energies for all samples varying in the peak position and ratio for the different particle structures. The two transition energies at 3.15 and 3.41 eV were found for the SD method, and 3.15 and 3.31 eV for SA and SG methods (Figure 9C and Supplementary Figure S10). The band gaps were determined using Tauc’s plot (Figure 9D) as 3.23 eV for SD and 3.10 eV for SA and SG methods. These values are in accordance with the reported values for the rutile and anatase phase of TiO_2_ (3.11 eV and 2.86—3.34 eV, respectively) (Lin et al., 2006). A slight decrease in band gap is observed for SA, SG, and dip deposition methods compared to SD. This is attributed to the amount of adsorbed water remaining on the surface of TiO_2_ agglomerates after the deposition process (Supplementary Figure S8). As shown by a theoretical study of Liu et al. (2016), the presence of adsorbed water on the surface of TiO_2_ acted as a weak electron donor leading to the valence band maximum upshifting, which resulted in the slight band narrowing of 0.2 eV. Similarly, the photoabsorption of reduced TiO_2_ was enhanced with the presence of adsorbed water in another study (Wen et al., 2018). Our findings suggest that the SA and SG deposition methods provided better photoabsorption not only due to the uniform distribution of TiO_2_ nanoparticles onto the glass fibers but also due to the presence of adsorbed water affecting the electronic surface structure.

### 3.2 Photocatalytic Performance of PCO Filters

**FIGURE 10:**
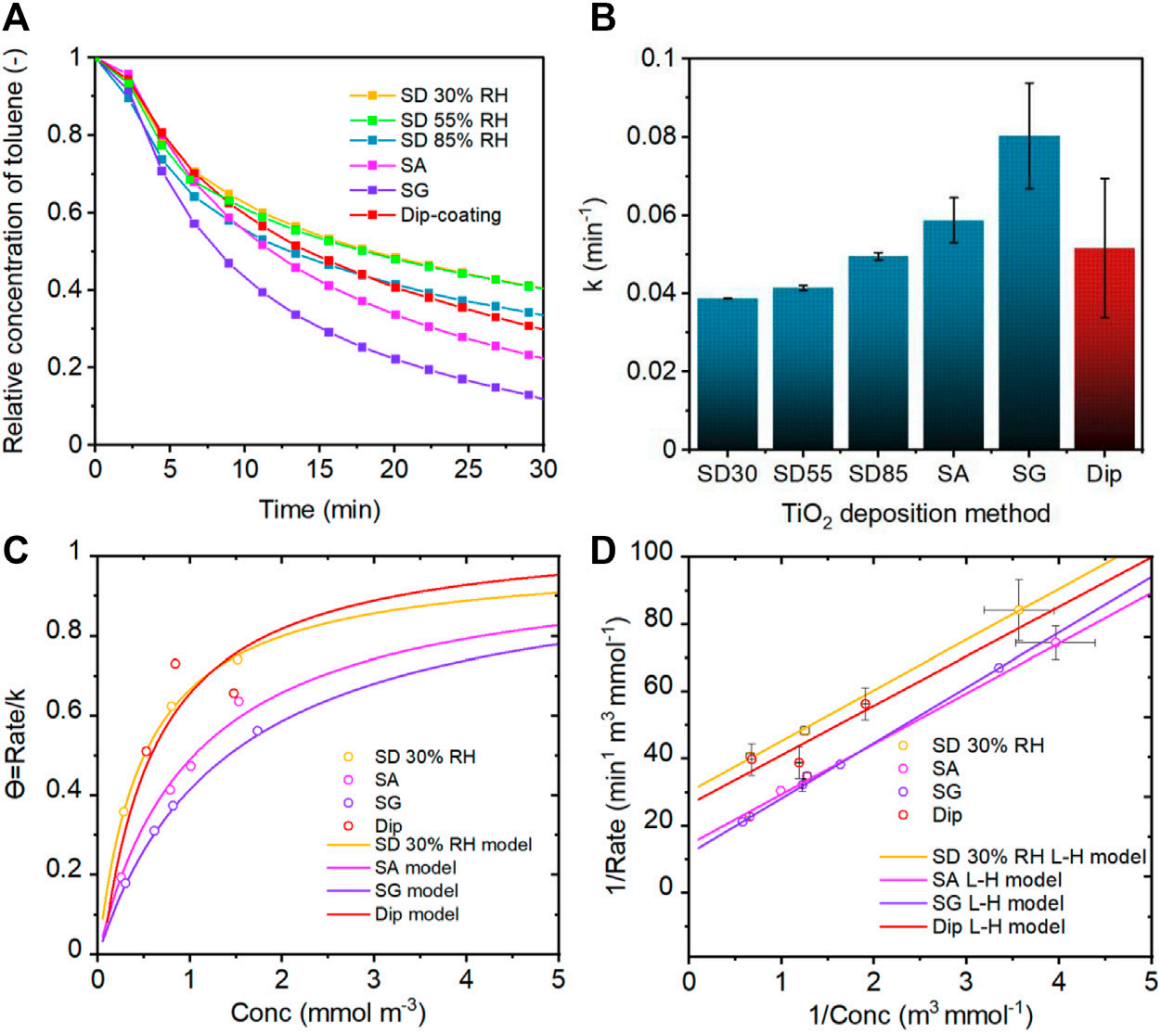
**(A)** UV-driven photocatalytic performance of TiO_2_-coated filters using spray-drying (SD), spray atomization (SA), and spray gun (SG) deposition methods in comparison with the dip-coating method. **(B)** Pseudo-first-order kinetic constant *k* dependence on the deposition method. L-H kinetic model analysis for **(C)** coverage and **(D)** photocatalytic activity of TiO_2_-coated filters prepared by SD 30%RH, SA, SG, and dip-coating.

The photocatalytic activity of TiO_2_-loaded filters prepared by different aerosol-assisted methods was evaluated using the pseudo-first-order kinetic and Langmuir–Hinshelwood (L-H) models (Liu et al., 2005; Debono et al., 2011; Kim et al., 2012). Toluene has been selected as a model pollutant for the evaluation as its photocatalytic degradation mechanism has been investigated extensively (Marcì et al., 2003; Sleiman et al., 2009; Bianchi et al., 2014; Chen et al., 2021). The results from the pseudo-first-order kinetic analysis showed that the photocatalytic activity depended on the preparation conditions of the photocatalytic filters, and the highest activity was achieved with the samples prepared by SA and SG methods (Figures 10A and B). The SG method showed the best photocatalytic activity followed by the SA method, whereas SD 30RH% revealed the worst activity. These methods were further evaluated using the L-H model.

The L-H model analysis revealed information about the adsorption properties and degradation rate of toluene. It was shown that the adsorption constant and site accessibility of coatings toward the toluene pollutant decreased in the order of SD 30%RH > SA > SG conditions (Supplementary Table S1). The decreased adsorption was also represented by the coverage Θ (Figure 10C). Although the adsorption of toluene on the TiO_2_ surface was greater for the SD method, the reaction rate was lower for SA and SG methods. This behavior was attributed to a strong competition between adsorbed water and pollutant (e.g., toluene) (Zhang et al., 2020). Therefore, the adsorbed water that remained on the sample surface after the preparation and drying process was analyzed using a TGA technique. The TGA analysis revealed that the SA and SG methods led to greater hydration remaining on the TiO_2_ surface (Figure 8B). As reported in several studies, adsorbed water can occupy the active sites and limit the accessibility of pollutant molecules when the amount of adsorbed water is high (Coronado et al., 2003; Jeong et al., 2013). On the other hand, the presence of water and hydroxyl groups promotes the formation of hydroxyl radicals, which are generally considered to initiate the degradation of adsorbed pollutants upon irradiation (Coronado et al., 2003; Park et al., 2012). Moreover, the adsorbed water on the TiO_2_ surface stabilizes photogenerated charge carriers and delays the electron–hole recombination compared to the dehydrated TiO_2,_ as shown by Litke et al. (2017). Furthermore, it was reported that the increasing amount of adsorbed water/hydroxyl groups enhanced the complete oxidation of toluene molecules (Jeong et al., 2013). The photocatalytic reaction is a complex process involving many steps and is affected by multiple factors. Based on our results, the enhanced degradation performance of toluene using SA and SG samples was attributed to the above-discussed hydration along with the structural features of TiO_2_ coating, for example, larger pore size and lower fractal dimension of agglomerates. The hydration provided the enrichment of the reactive species formation (i.e., hydroxyl radicals) during the irradiation of TiO_2,_ while the structural features enabled efficient irradiation (Brosillon et al., 2008; He et al., 2014; Sun et al., 2018). In our study, irradiation was not the limiting factor for the photocatalytic activity of TiO_2_ deposited by aerosol-assisted methods. As shown by the optical properties of prepared samples (Figure 9), the sharp change in the UV absorption between SD and SA/SG methods does not reflect the continuous increase in the reaction rate (Figure 10B). Therefore, the superior reaction rate of SA and SG samples is mainly attributed to the synergistic effect of the agglomerate and pore properties together with the amount of remaining water molecules on the surface/in the pores of TiO_2_ agglomerates. Specifically, for SD 30%RH, the amount of water and pore size and volume was small, and thus, the amount of generated radicals was the most limited. As the water remained on the surface and/or into pores due to the SA and SG preparation conditions, the formation of reactive species and slower electron–hole recombination enhanced the degradation efficiency of toluene. These findings are consistent with the study of He et al. (2015), who highlighted the importance of pore size and wide pore size distribution for the photocatalytic activity of mesoporous TiO_2_. The large pore size distribution enhances the accessibility of small and otherwise separated pores and therefore facilitates the adsorption and competition of molecules, efficient light activation, and reactive species generation (He et al., 2015). On the other hand, the dip-coating method showed similar toluene adsorption behavior and coverage as SD 30%RH but slightly higher photocatalytic performance. This is assigned to the formation of a thick layer of TiO_2_ NPs during the dip-coating process. Similarly, as for the SD method, the thick agglomerates were located on the top of filter fibers, which allowed higher adsorption of the pollutant but limited the photoabsorption and efficient activation. Although the photoabsorption was relatively high due to the low reflection, the photocatalytic activity was reduced in comparison to SA and SG methods owing to the loss of the incident light due to the transmission through the uncoated parts of the filter (Supplementary Figure S10).

### 3.3 Photocatalytic Coating Quality Factor

For the practical implementation of photocatalytically active filters, the choice of the photocatalyst immobilization procedure would affect not only the removal performance of toluene but also the operational and mechanical properties, such as permeability of the filter (and pressure drop) and mechanical stability of TiO_2_ coating. Therefore, we propose a simple evaluation of the overall PCO filter performance using a parameter entitled “coating quality factor”, which is based on the application of a broadly used concept to assess the filtration efficiency (Wang et al., 2008). This evaluation combines parameters, such as the reaction constant k (min^−1^), the permeability of filter K (m^2^), and mass loss of coatings (g m^−2^) as follows:

$$"Coating\;Quality\;Factor"=\frac{k.K}{m_{loss}}\left(m^{4}.g^{-1}.min^{-1}\right).$$ (6)

**FIGURE 11:**
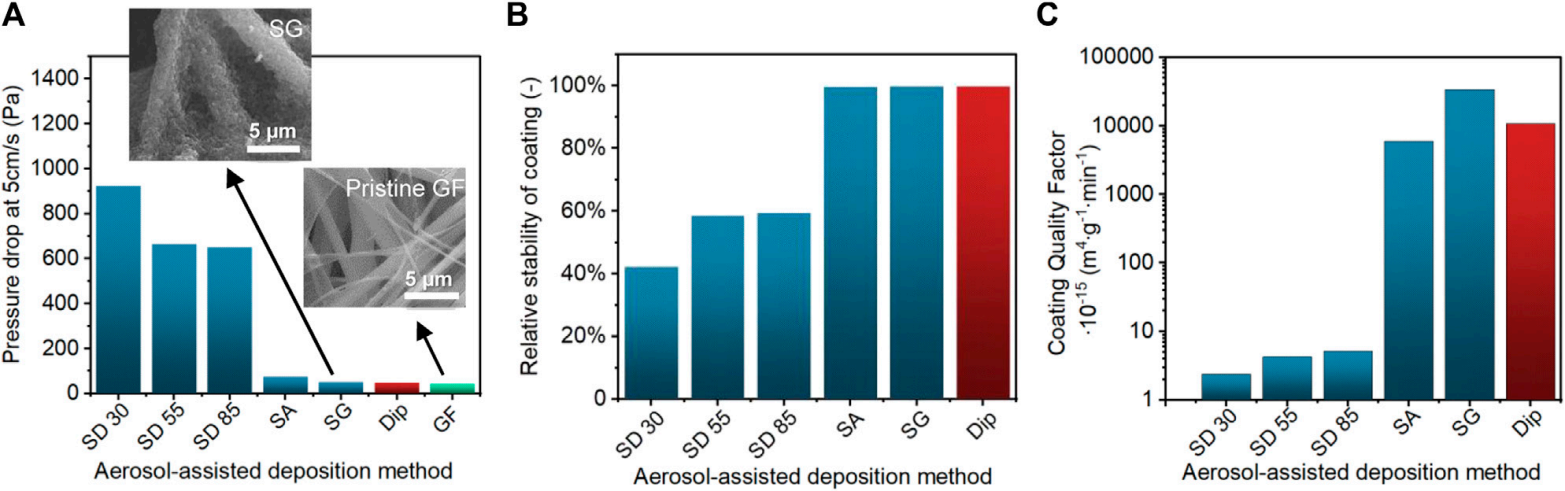
**(A)** Pressure drop across the coated filters determined at the air velocity of 5 cm s^−1^ with SEM images of pristine glass fibers (GF) and coated glass fibers by the SG method. **(B)** Relative stability of TiO_2_ coatings after an applied ultrasonic stress. **(C)** Coating quality factor of TiO_2_ coated filters determined by VOC degradation constant (min^−1^) and permeability (m^2^) divided by TiO_2_ loss during stability test (g m^−2^).

It provides a quantitative comparison of PCO filters including reactivity and operational properties. The evaluation of pressure drop (Figure 11A) was performed at a standard value of 5 cm s^−1^ for filtration studies, which exceeded significantly the filtration velocities used for the PCO tests. It, nevertheless, provides comparable information about the permeability *K* of the airflow through the filters tested at the same velocity. The stability of coating (Figure 11B) (i.e., loss of coating under applied stress m_loss_) provides another important information for the practical implementation, which is the adhesion of photocatalyst particles onto the filter tested under ultrasonic stress. The SD methods demonstrated both large pressure drop and poor stability of TiO_2_ coating. This was caused by the formation of “a cake” between and on the top of filter fibers. On the other hand, SA and SG methods displayed low-pressure drop and high stability of coating (Figures 11B and C) as TiO_2_ nanoparticles formed a continuous layer wrapping around each individual fiber. The thinner coating layer provided better adhesion strength than the SD method. The van der Waals attraction forces between the TiO_2_ agglomerates and GF substrate showed better adhesion strength and facilitated the stability of SA coating, while these forces were weaker in the presence of the large and round agglomerates produced by the SD method (Walsh et al., 2012; Gardon and Guilemany, 2014). As a result, the obtained “coating quality factor” values differ significantly for SA and SG conditions in comparison to the SD method (Figure 11C). The microstructure and macrostructure formed during the deposition using the dispersion of nanoparticle suspension through an atomization process (SA and SG) demonstrated both enhanced PCO reactivity and improved operational performance. The best overall performance was obtained for the SG method due to the larger droplet size and higher deposition speed. The benchmark dip-coating method demonstrated comparable performance with SA and SG methods. Even though the SA method displayed slightly lower overall performance than SG and dip-coating methods, this method has a potential for further optimization and improvement due to its advantages. First, the deposition conditions can be controlled efficiently (resulting in lower deviations in the performances). Second, it can be operated as a continuous deposition process resulting in better control of mass loading, that is, eliminating the need for the multiple immobilization and drying steps. Despite the high photocatalytic performance of TiO_2_ photocatalyst, its efficiency has been reduced in the cycle tests due to the photocatalyst poisoning effect (Supplementary Figure S11) (Weon et al., 2018). Nevertheless, several strategies have been employed to minimize the damage, for example, catalyst doping and the control of catalyst properties, process, and regeneration conditions (Wu et al., 2021). Hence, these strategies could be integrated with SA and/or SG deposition methods in future studies. Additionally, these deposition methods provide many possibilities for the adjustment of deposition conditions, such as the concentration and pH of TiO_2_ suspension (Wang et al., 2005; Sen et al., 2009; Lee et al., 2010) and/or the addition of surfactants (Veronovski et al., 2010). These adjustments would enable further investigation of the agglomeration and deposition properties and their effect on the photocatalytic performance. Furthermore, different types of nozzles could be selected and evaluated (Lee et al., 2011).

## 4 Conclusion

Spray-drying (SD), spray atomization (SA), and spray gun (SG) methods were applied for the immobilization of TiO_2_ nanoparticles onto glass fiber filter media. The effects of the three different aerosol-assisted deposition techniques on the photocatalytic and operational performance were evaluated through the characterization of the structural and optical properties, hydration, coating stability, and toluene degradation activity. The SD method resulted in the deposition of compact and round agglomerates possessing limited mechanical stability of the coating and reduced photocatalytic activity. In contrast, SA and SG methods formed a uniform film of TiO_2_ nanoparticles on the individual glass fibers. This was enabled by the deposition of droplet dispersions containing open and fractal-like agglomerated nanoparticles. The resulted coating structures provided good mechanical stability and sufficient exposure to the incident light followed by efficient activation of the TiO_2_ photocatalyst. In addition, the pore characteristics together with the remaining hydration on the surface of TiO_2_ resulted in the enhancement of photocatalytic activity through the generation of reactive species instrumental for the oxidation of toluene. Our findings demonstrate that the SA and SG methods could be promising alternatives to the benchmark dip-coating method. In particular, the SA method enables a continuous deposition process demonstrating better control of the deposition conditions and mass loading, which offers potential for further optimization and improvement of photocatalytic performance.

## Data Availability

The original contributions presented in the study are included in the article/Supplementary Material, further inquiries can be directed to the corresponding author.
