# Supplementary material for: Aerosol-Assisted Deposition for TiO2 Immobilization on Photocatalytic Fibrous Filters for VOC Degradation
Source: Front Chem. 2022 May 11;10:887431. doi: 10.3389/fchem.2022.887431 (PMC9130724; doi:10.3389/fchem.2022.887431)
Supplement: Supplementary file 1 [file DataSheet1.PDF]

Supplementary Material

1 Supplementary Text

Effect of deposition method on TiO_2 agglomerate structure

The observed structures of TiO_2 agglomeration resulted from the mechanism behind the atomization, deposition and drying processes. After atomization, the particles were reorganized to offset the capillary forces caused by the condensation and evaporation of the liquid bridges between the TiO_2 NPs in the form of colloidal droplets. These forces were favoured between the hydrophilic surfaces of TiO_2 that expedited water adsorption. In the case of SA and SG, the colloidal droplets were deposited directly on the substrate with subsequent evaporation of water in ambient conditions allowing the uniform dispersion and coverage of GF substrate. On the other hand, in the SD preparation method, the colloidal droplets evaporated rapidly already in the air stream controlled by RH conditions, which affected the shape and size of the deposited TiO_2 nanoparticles. The rate of drying was fast enough to weaken the forces that hold the liquid bridges together and the particles collapsed from micro-sized droplets into round and more compact sub-micro structures. The rate of drying during the SD process can be represented by the Peclet number (Pe), which is determined as the ratio of R^2 and $D\tau$ where R is the radius of droplet, D is the diffusion coefficient of colloid particles in the droplet, and τ is the time of drying. In our case, the time of drying was determined from the air velocity and the dimension of drying tube as 0.06 s and the number median diameter of droplets from the TSI atomizer was 0.3 µm. The diffusion coefficient ($3.1315\cdot10^{-11}$ m^2/s) was calculated from Einstein-Stokes relation, as shown the study of Sen et al. (Sen et al., 2009). The result Peclet number was 0.012, which might be considered as intermediate to rather slow drying rate ($Pe \ll 1$). The intermediate drying rate has been reported to form isotonic shrinkage of silica (Lee et al., 2010) and alumina particles (Sen et al., 2009), i.e. without the formation of viscoelastic shell of densely packed particles. It is noteworthy to mention that the shrinkage and final morphology of grains is influenced not only by the drying rate, but also the concentration and structure of particles inside the droplet (Sen et al., 2009; Bahadur et al., 2011), hydrodynamics of drying (Wang et al., 2005), and surface tension. Wang et al. (Wang et al., 2005) investigated the dispersion, evaporation of colloidal droplets, and the aggregation of nanoparticles. According to their study, the formation of hard round agglomerates could be explained by the effect of Laplace and osmotic pressure. The Laplace pressure tended to keep the droplet spherical and increased during the evaporation stage of spray-drying process in order to minimize droplet`s free energy. If the osmotic pressure within the droplet was smaller than Laplace pressure, the particles were forced to be reorganized, and the droplet would shrink and hard aggregates would form (Zeng and Weber, 2014).

2 Supplementary Figures and Tables

2.1 Supplementary Figures

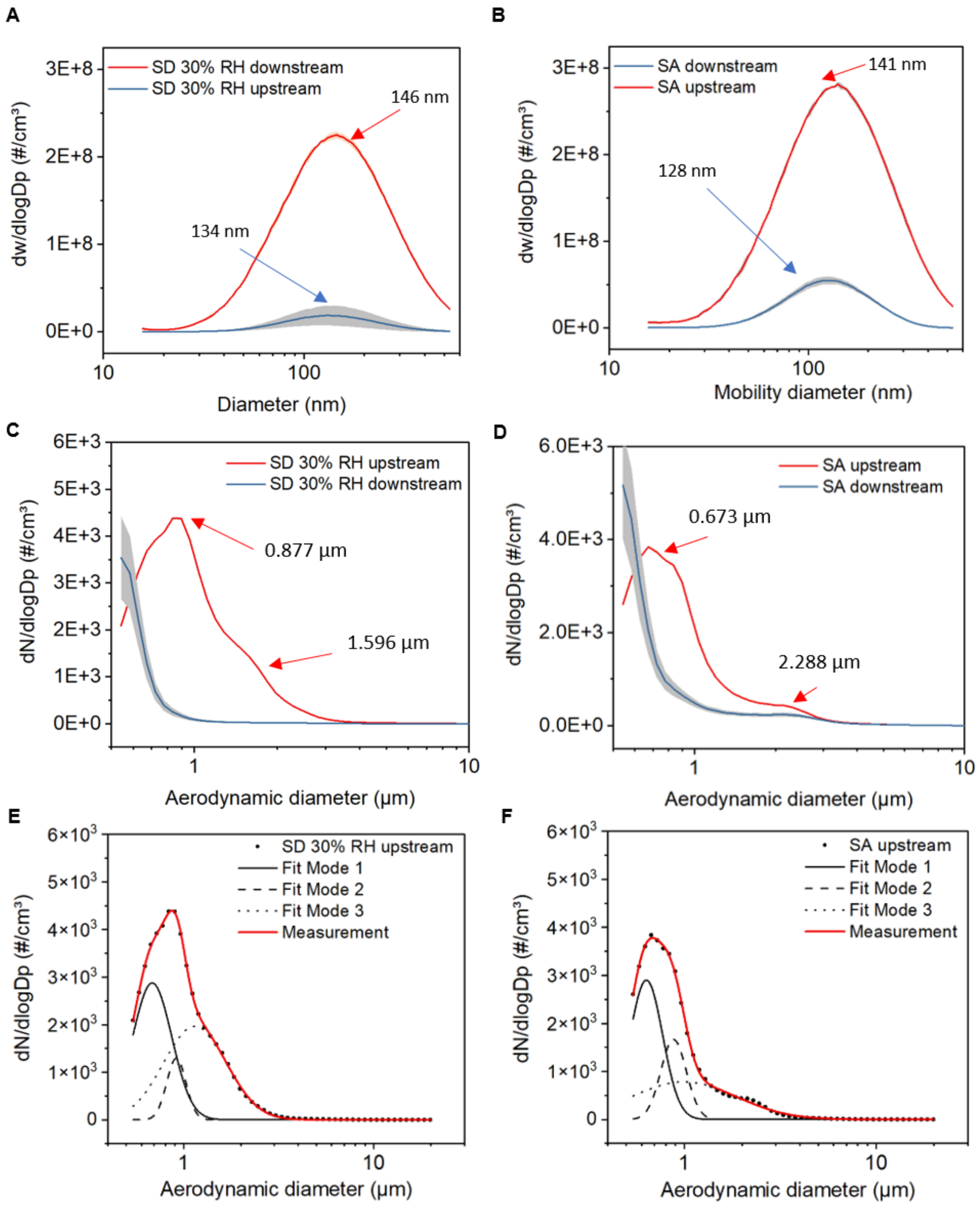

Figure S1 Size distribution of atomized TiO_2 particles using **(A)** and **(B)** scanning mobility particle sizer (SMPS) and **(C)** and **(D)** aerodynamic particle sizer (APS) in upstream and downstream of SD 30%RH and SA deposition conditions. The size of particles atomized under SA conditions is slightly

smaller than the size of particles atomized under SD 30%RH. It should be noted, however, that the droplets generated with the SA method are died out in the sheath air stream of SMPS analyser. Therefore, the size distribution may not represent the real size of agglomerates deposited on the filter media. Lognormal particle size distribution from SMPS data revealed Count Mode Diameter (CMD) of 146 nm and 141 nm for SD and SA method, respectively. APS analysis revealed three-modal size distributions for **(E)** SD and **(F)** SA method with CMD of 0.680 μm, 0.869 μm, and 0.917 μm for SD, and with CMD of 0.635 μm, 0.881 μm, and 0.996 for SA method.

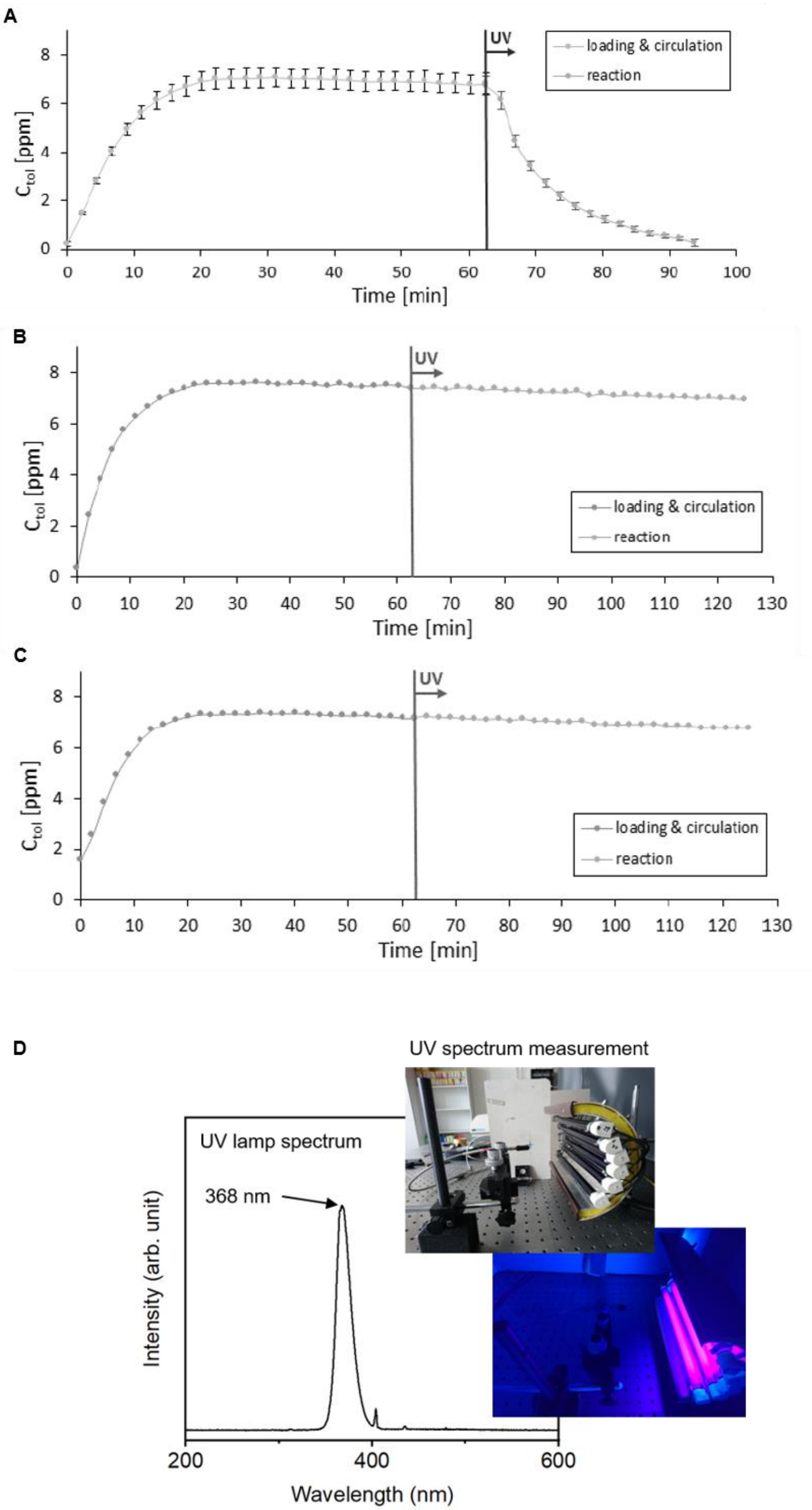
A
UV
loading & circulation
reaction
C_{tol} [ppm]
Time [min]
B
UV
loading & circulation
reaction
C_{tol} [ppm]
Time [min]
C
UV
loading & circulation
reaction
C_{tol} [ppm]
Time [min]
D
UV spectrum measurement
UV lamp spectrum
368 nm
Intensity (arb. unit)
Wavelength (nm)

Figure S2 (A) Photocatalytic reaction representation (coated filter + toluene), **(B)** evaluation of substrate effect (uncoated filter + toluene), **(C)** and evaluation of photolysis (empty reactor + toluene) throughout the loading, circulation and irradiation stages, for 5 ppm of toluene. **(D)** UV light spectrum of the used lamp (6x8W BLB bulbs from Philips) measured using HR 4000 spectrometer (200nm – 1000nm) from Ocean Optics.

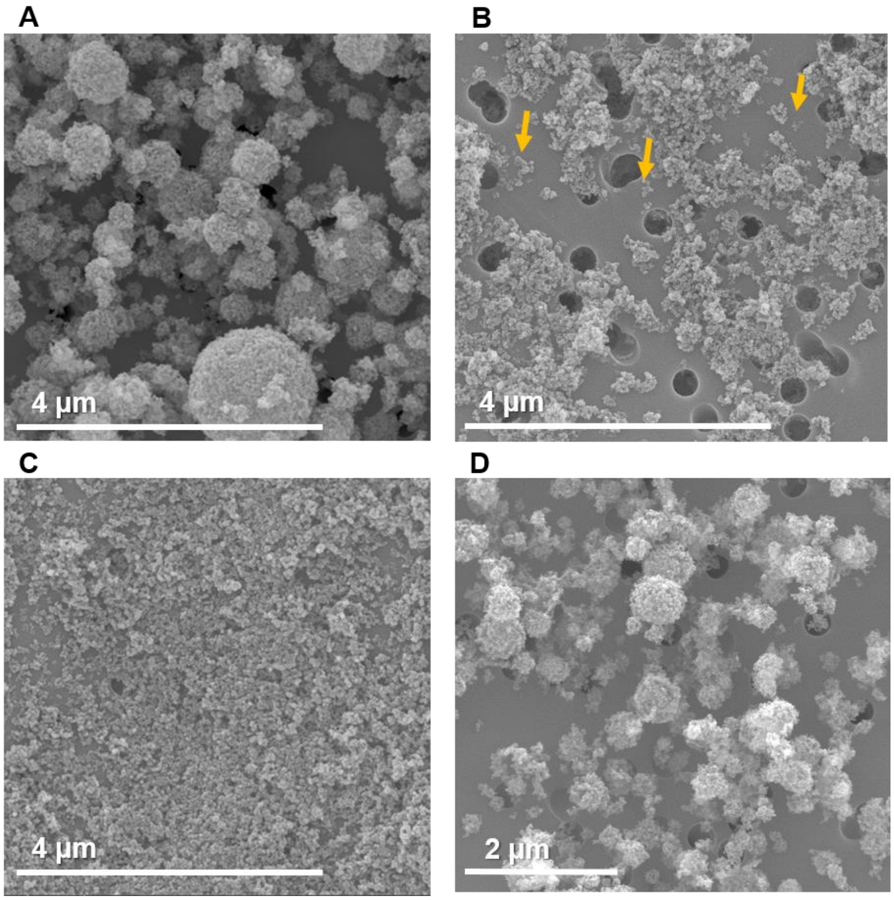

Figure S3 SEM images of **(A)** SD 55%RH, **(B)** SA deposited TiO_2 agglomerates onto flat Nucleopore filter with highlighted fragmented structures, **(C)** SG to illustrate the fragmentation of agglomerates prepared under higher RH conditions. **(D)** Appearance of round agglomerates after humidification using humidifier demonstrating the presence of hard agglomerates

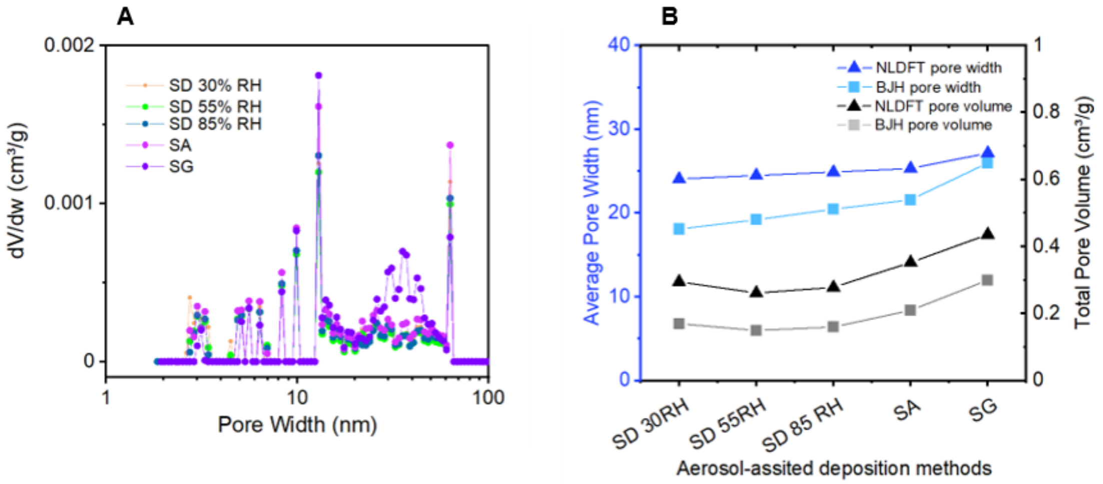

Figure S4 (A) NLDFT pore size distribution for different aerosol-assisted deposition methods. **(B)** Average pore width and total pore volume determined by BJH and NLDFT model.

A

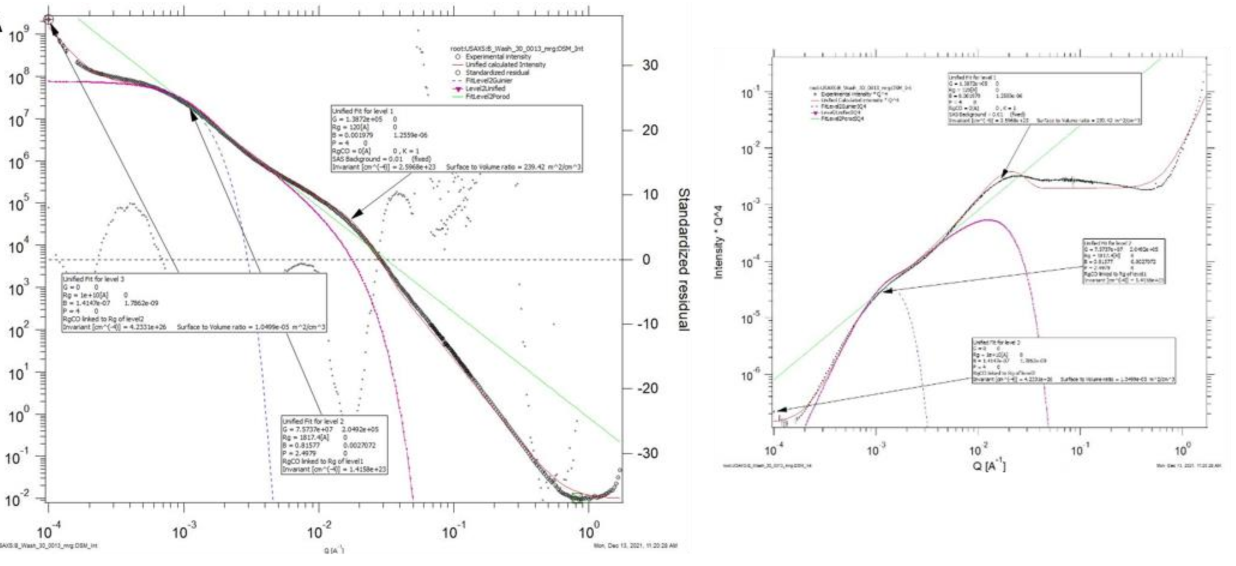
Standardized residual
Intensity * Q^4
Q [A^-1]

B

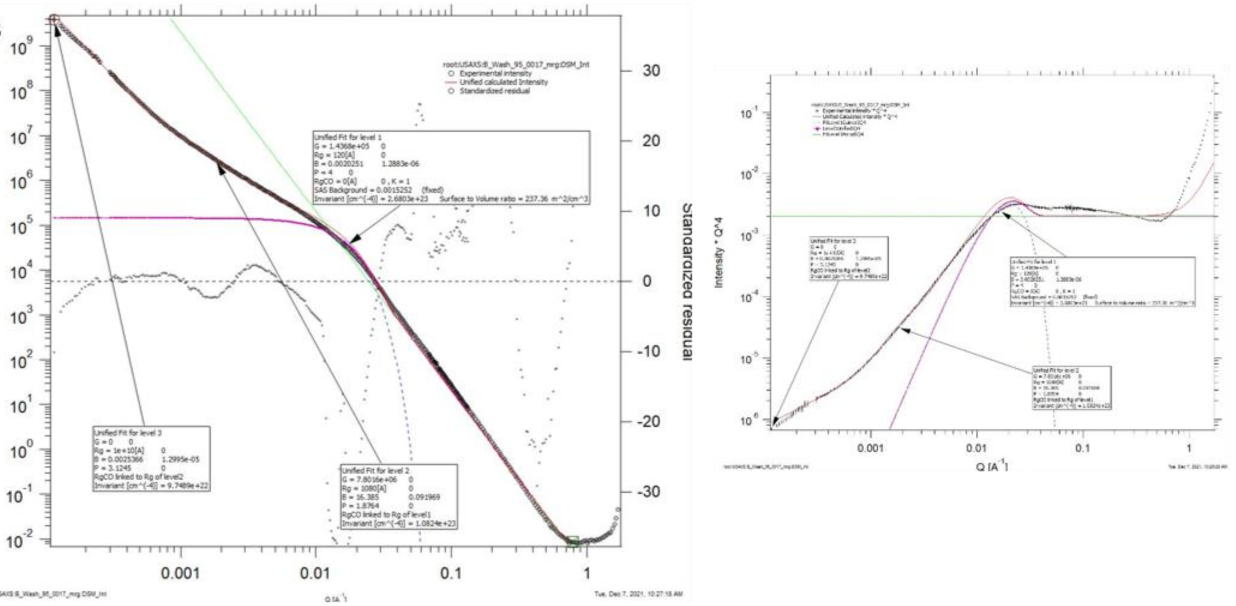
Standardized residual
Intensity * Q^4
Q [A^-1]

C

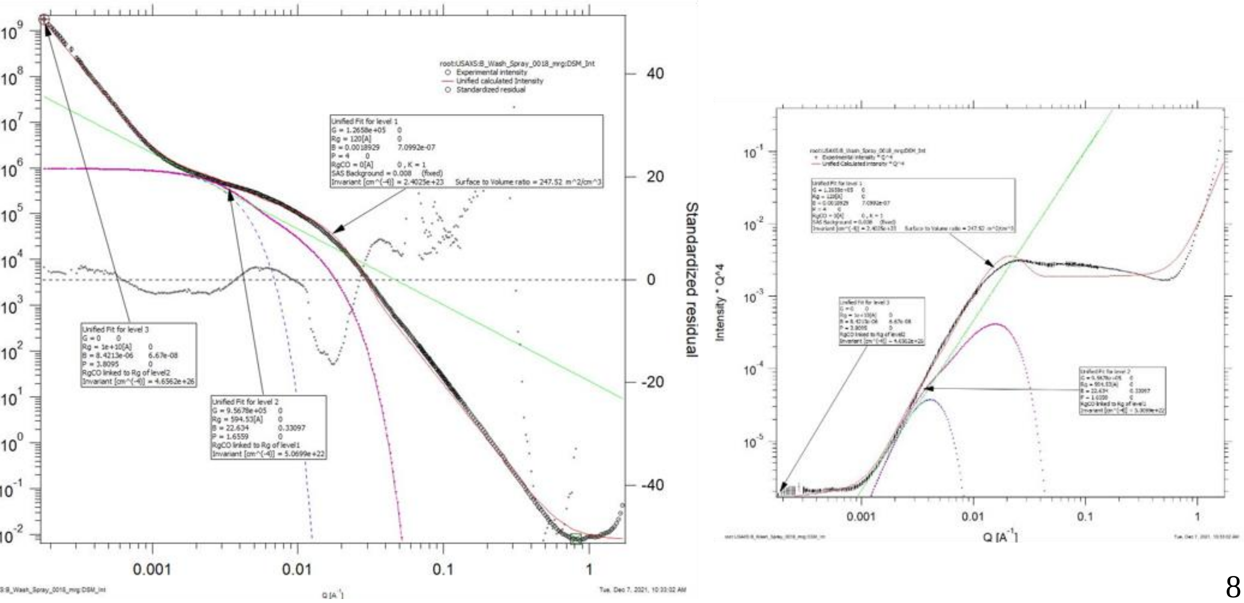
Standardized residual
Intensity * Q^4
Q [A^-1]

Figure S5 USAXS data with two levels composed of a Guinier parts and a power law tails fitted with Irena Unified fit code for **(A)** SD 30%RH, **(B)** SA and **(C)** SG. For the 1st level, the radius of gyration R_{g1} was adjusted according to the primary particles radius known for TiO_2 P25 and it was fitted with fixed value of 120 Å for all the samples. The primary particles were assumed to be smooth, and thus the slope of Porod regime was fixed to 4. The R_g of second level was linked to R_g of 1st level as the agglomerates consisted of the primary particles of TiO_2. The resulted P value of 2nd level fit was assigned to the fractal dimensions of agglomerates. The 3rd level (low Q regime) was fitted setting G parameter to 0 and P value to 4 and fitting the parameter B because this surface structural level corresponding to the large object was not completely coved by the measurement, and thus, no conclusions can be driven from this region.

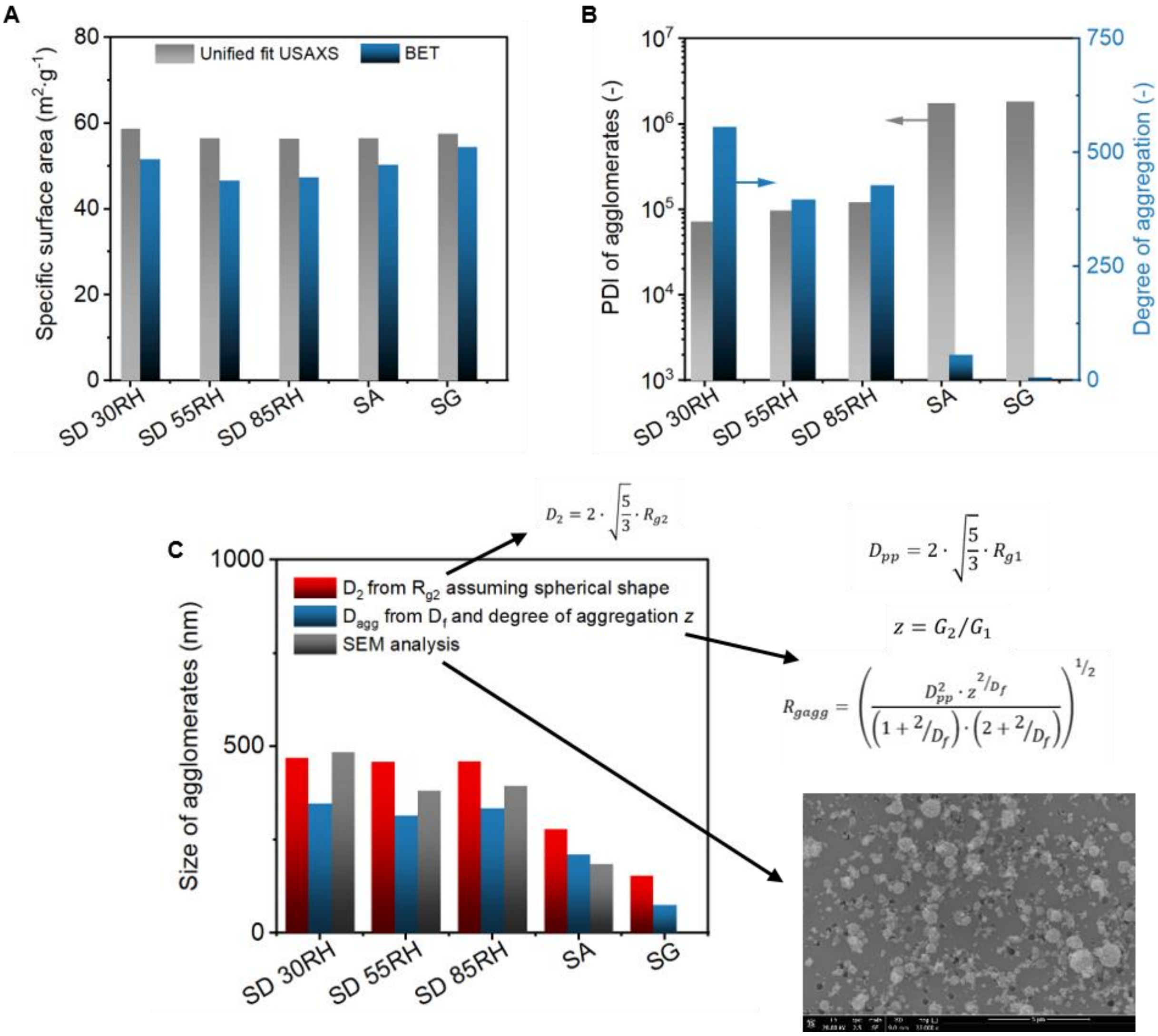

Figure S6 (A) Comparison of specific surface areas (SSA) obtained from Unified fit and BET method. The Unified fit provided higher SSA by from 8% to 15%, which is within the range of reported values for TiO_2 P25 by BET method (from 50 $m^2 \cdot g^{-1}$ to 60 $m^2 \cdot g^{-1}$) (Wang et al., 2012; Colombo et al., 2017). **(B)** Index of polydispersity PDI for agglomerates formed during different methods calculated from the parameters determined from Unified fit as PDI = $BR_{g2}^4/(1.62G)$ (right y-axis) and degree of aggregation *z* calculated by as a ratio of G2 and G1 of Unified fit (left y-axis). PDI is a single parameter that can be directly applied to the mass-fractal aggregate scattering of a series of samples regardless of changes in particle shape and distribution functions (Beaucage et al., 2004). The formation of open agglomerates together with fragmentation and reorganisation resulted in the increase of PDI. **(C)** Comparison of agglomerate sizes determined by three different methods. First, the red bars correspond to the diameter calculated from radius of gyration from level 2 Unified fit R_{g2} assuming spherical shape. Second, the blue bars display the diameter calculated as $2 \cdot R_{agg}$ including the diameter of primary particles, degree of aggregation and fractal dimensions (all parameters obtained from Unified fit). Third, the grey bars represent the mean diameter determined from the normal size distribution of separated agglomerates imaged by SEM on Nucleopore filters. It

should be noted that the SEM analysis of the SG sample is not provided due to the difficulty in obtaining and distinguishing the separated agglomerates in the SEM image.

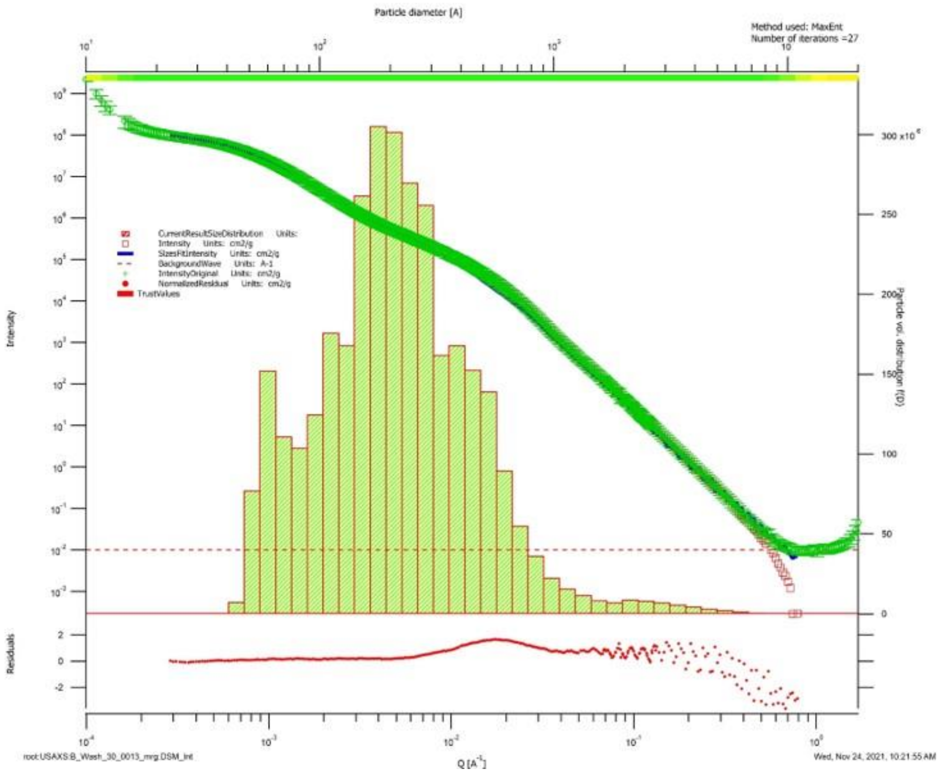

Figure S7 Size distribution of USAXS curve using maximum entropy method in Irena tool for SD 30% RH sample displaying histogram of size volume distribution (top x-axis and right y-axis), USAX curve fit applied a user % error (bottom x-axis and left y-axis), and residues.

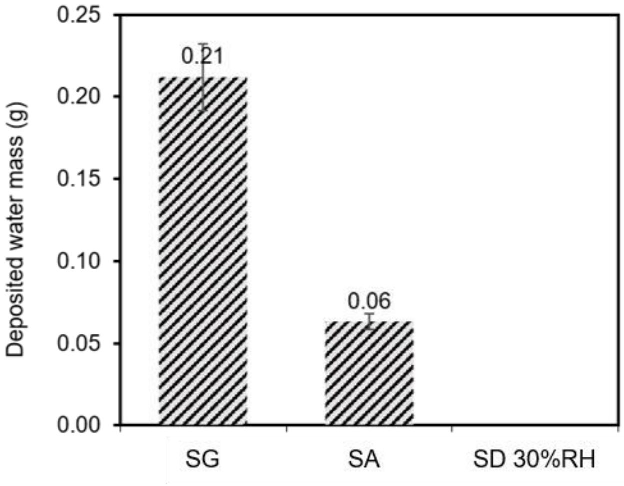

Figure S8 Deposited water determined by mass of the filter after coating process under SG, SA and SD 30% RH condition and after the completed drying.

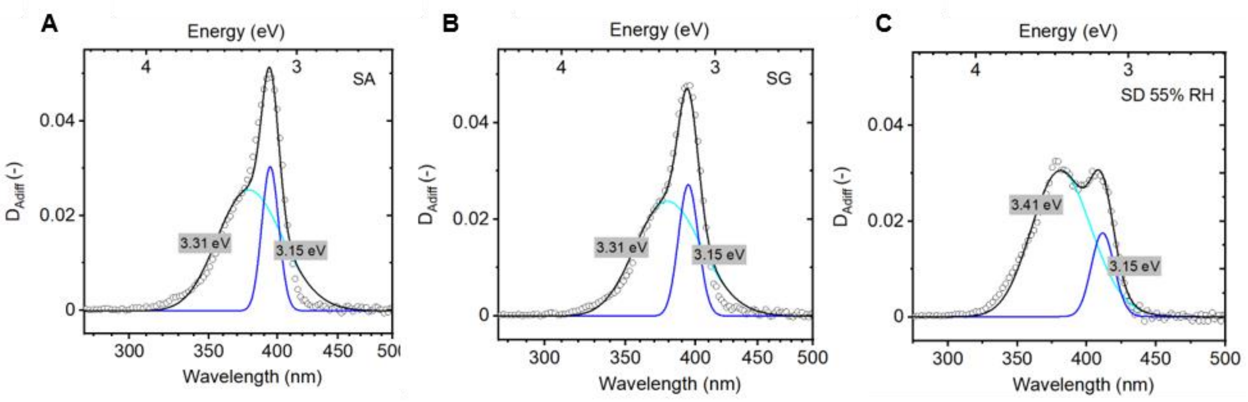

Figure S9 First derivative of absorption spectra for **(A)** SA, **(B)** SG and **(C)** SD 55% RH deposition method.

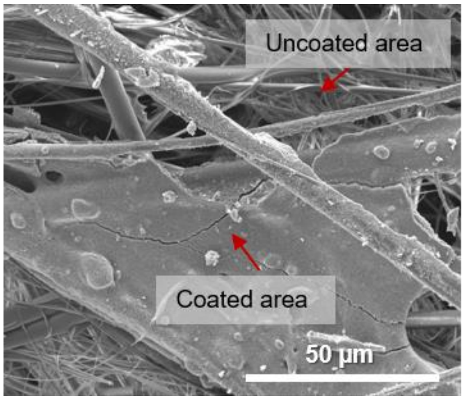

Figure S10 SEM image of TiO_2 coated glass fibre filter coated by dip-coating method with representative coated and uncoated areas (red arrows).

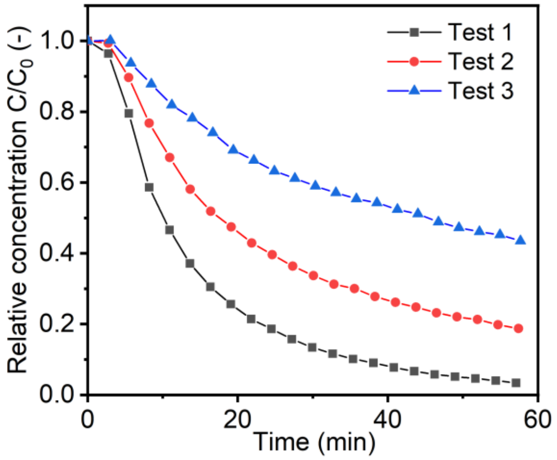

Figure S11 Photocatalytic stability of dip-coated sample over three subsequent photocatalytic tests. The photocatalytic activity was reduced by 16% and 34% in the second and third cycle. Our findings are in the agreement with the study of Weon et al. where the deactivation of almost 37% and 55% in the second and third cycle, respectively, has been reported during toluene degradation using benchmarked TiO_2 photocatalyst (Weon et al., 2018).

2.2 Supplementary Tables

Table S1 Summary of obtained results

	30% RH TiO_2	55% RH TiO_2	85% RH TiO_2	95% RH TiO_2	Spray TiO_2
BET surface area, S_{BET} ($m^2 \cdot g^{-1}$)	51.6	46.6	47.4	50.4	54.4
Pore volume BJH[a], V_{pore} ($cm^3 \cdot g^{-1}$)	0.17	0.15	0.16	0.21	0.30
Pore volume NLDFT, V_{pore} ($cm^3 \cdot g^{-1}$)	0.30	0.26	0.28	0.35	0.44
Pore size BJH[a] (nm)	18.1	19.2	20.5	21.6	26.0
Pore size NLDFT (nm)	24.1	24.5	24.9	25.3	27.2
Crystal size[b] (nm)	24.2 ± 1.8	24.3 ± 1.9	26.6 ± 2.2	22.7 ± 1.6	24.4 ± 1.9
Density[c] ($g \cdot cm^{-3}$)	4.15	4.05	4.11	4.08	3.80
Porosity (%)	7	-	-	6	4
USAXS diameter of agglomerates D_2 (nm)	469	458	460	279	154
USAXS fractal dimension D_f (-)	2.5	2.5	2.4	1.9	1.7
USAXS specific surface area[d] ($m^2 \cdot g^{-1}$)	59	56	56	57	58
1st order kinetic constant k_1 (min^{-1})	0.040 ± 0.002	0.041 ± 0.001	0.050 ± 0.001	0.056 ± 0.002	0.079 ± 0.006
Normalized k_1 ($g \cdot m^{-2} \cdot min^{-1}$)	0.0008	0.0009	0.0011	0.0011	0.0015
L-H kinetic reaction constant $k_{L\text{-}H}$ ($m^3 \cdot mmol^{-1} \cdot min^{-1}$)	0.033	-	-	0.069	0.085
L-H adsorption constant $K_{L\text{-}H}$ ($m^3 \cdot mmol^{-1}$)	2.001	-	-	0.963	0.710
Stability (%)	42%	58%	59%	100%	100%
Pressure drop at 5 $cm \cdot s^{-1}$ (Pa)	924 ± 136	665 ± 98	650	81 ± 6	52 ± 0.3
Permeability K (m^2)	4.1	5.2	5.2	42.2	64.4
"Coating quality factor" ($m^4 \cdot min^{-1}$)	7	13	15	236	508

[a] Desorption BJH. [b]Determined by Scherrer equation from XRD analysis – average of XRD peaks. [c]Density of powder samples using pycnometry. [d] Calculated as surface to volume in $m^2 \cdot cm^{-3}$ divided by the bulk density of TiO_2 (4.2 $g \cdot cm^{-3}$).

3 Supplementary References

Bahadur, J., Sen, D., Mazumder, S., Bhattacharya, S., Frielinghaus, H., and Goerigk, G. (2011). Origin of Buckling Phenomenon during Drying of Micrometer-Sized Colloidal Droplets. *Langmuir* 27(13)**,** 8404-8414. doi: 10.1021/la200827n.

Beaucage, G., Kammler, H.K., and Pratsinis, S.E. (2004). Particle size distributions from small-angle scattering using global scattering functions. *Journal of Applied Crystallography* 37(4)**,** 523-535. doi: doi:10.1107/S0021889804008969.

Colombo, E., Li, W., Bhangu, S.K., and Ashokkumar, M. (2017). Chitosan microspheres as a template for TiO2 and ZnO microparticles: studies on mechanism, functionalization and applications in photocatalysis and H2S removal. *RSC Advances* 7(31)**,** 19373-19383. doi: 10.1039/C7RA01227F.

Lee, S.Y., Gradon, L., Janeczko, S., Iskandar, F., and Okuyama, K. (2010). Formation of Highly Ordered Nanostructures by Drying Micrometer Colloidal Droplets. *ACS Nano* 4(8)**,** 4717-4724. doi: 10.1021/nn101297c.

Sen, D., Mazumder, S., Melo, J.S., Khan, A., Bhattyacharya, S., and D'Souza, S.F. (2009). Evaporation Driven Self-Assembly of a Colloidal Dispersion during Spray Drying: Volume Fraction Dependent Morphological Transition. *Langmuir* 25(12)**,** 6690-6695. doi: 10.1021/la900160z.

Wang, W.-N., Lenggoro, I.W., and Okuyama, K. (2005). Dispersion and aggregation of nanoparticles derived from colloidal droplets under low-pressure conditions. *Journal of Colloid and Interface Science* 288(2)**,** 423-431. doi: https://doi.org/10.1016/j.jcis.2005.03.010.

Wang, X., Pehkonen, S.O., Rämö, J., Väänänen, M., Highfield, J.G., and Laasonen, K. (2012). Experimental and computational studies of nitrogen doped Degussa P25 TiO2: application to visible-light driven photo-oxidation of As(iii). *Catalysis Science & Technology* 2(4)**,** 784-793. doi: 10.1039/C2CY00486K.

Weon, S., Kim, J., and Choi, W. (2018). Dual-components modified TiO2 with Pt and fluoride as deactivation-resistant photocatalyst for the degradation of volatile organic compound. *Applied Catalysis B: Environmental* 220**,** 1-8.

Zeng, L., and Weber, A.P. (2014). Aerosol synthesis of nanoporous silica particles with controlled pore size distribution. *Journal of Aerosol Science* 76**,** 1-12. doi: https://doi.org/10.1016/j.jaerosci.2014.05.003.
